# Genetic Ablation of G Protein-Gated Inwardly Rectifying K^+^ Channels Prevents Training-Induced Sinus Bradycardia

**DOI:** 10.3389/fphys.2020.519382

**Published:** 2021-01-20

**Authors:** Isabelle Bidaud, Alicia D’Souza, Gabriella Forte, Eleonora Torre, Denis Greuet, Steeve Thirard, Cali Anderson, Antony Chung You Chong, Angelo G. Torrente, Julien Roussel, Kevin Wickman, Mark R. Boyett, Matteo E. Mangoni, Pietro Mesirca

**Affiliations:** ^1^Institut de Génomique Fonctionnelle, Université de Montpellier, CNRS, INSERM, Montpellier, France; ^2^LabEx Ion Channels Science and Therapeutics, Montpellier, France; ^3^Division of Cardiovascular Sciences, University of Manchester, Manchester, United Kingdom; ^4^Department of Pharmacology, University of Minnesota, Minneapolis, MN, United States; ^5^Division of Biomedical Sciences, University of Copenhagen, Copenhagen, Denmark

**Keywords:** G-protein-gated inwardly rectifying potassium 4 (Girk4), hyperpolarization-activated cyclic nucleotide-gated 4 (HCN4) channel, bradycardia, sinoatrial node, endurance athletes

## Abstract

**Background:** Endurance athletes are prone to bradyarrhythmias, which in the long-term may underscore the increased incidence of pacemaker implantation reported in this population. Our previous work in rodent models has shown training-induced sinus bradycardia to be due to microRNA (miR)-mediated transcriptional remodeling of the HCN4 channel, leading to a reduction of the “funny” (*I*_f_) current in the sinoatrial node (SAN).

**Objective:** To test if genetic ablation of G-protein-gated inwardly rectifying potassium channel, also known as *I*_*KACh*_ channels prevents sinus bradycardia induced by intensive exercise training in mice.

**Methods:** Control wild-type (WT) and mice lacking GIRK4 (*Girk4*^–/–^), an integral subunit of *I*_*KACh*_ were assigned to trained or sedentary groups. Mice in the trained group underwent 1-h exercise swimming twice a day for 28 days, 7 days per week. We performed electrocardiogram recordings and echocardiography in both groups at baseline, during and after the training period. At training cessation, mice were euthanized and SAN tissues were isolated for patch clamp recordings in isolated SAN cells and molecular profiling by quantitative PCR (qPCR) and western blotting.

**Results:** At swimming cessation trained WT mice presented with a significantly lower resting HR that was reversible by acute *I*_*KACh*_ block whereas *Girk4*^–/–^ mice failed to develop a training-induced sinus bradycardia. In line with HR reduction, action potential rate, density of *I*_f_, as well as of T- and L-type Ca^2+^ currents (*I*_*CaT*_ and *I*_*CaL*_) were significantly reduced only in SAN cells obtained from WT-trained mice. *I*_f_ reduction in WT mice was concomitant with downregulation of HCN4 transcript and protein, attributable to increased expression of corresponding repressor microRNAs (miRs) whereas reduced *I*_*CaL*_ in WT mice was associated with reduced Ca_v_1.3 protein levels. Strikingly, *I*_*KACh*_ ablation suppressed all training-induced molecular remodeling observed in WT mice.

**Conclusion:** Genetic ablation of cardiac *I*_*KACh*_ in mice prevents exercise-induced sinus bradycardia by suppressing training induced remodeling of inward currents *I*_f_, *I*_*CaT*_ and *I*_*CaL*_ due in part to the prevention of miR-mediated transcriptional remodeling of HCN4 and likely post transcriptional remodeling of Ca_v_1.3. Strategies targeting cardiac *I*_*KACh*_ may therefore represent an alternative to pacemaker implantation for bradyarrhythmias seen in some veteran athletes.

## Introduction

The pacemaker activity of sinoatrial node (SAN) permanently controls the heart rate (HR) in everyday life (Mangoni and Nargeot, 2008). SAN pacemaking is generated by diastolic depolarization, a slow depolarizing phase of the action potential driving the membrane voltage from the end of the repolarization phase of the preceding action potential to the threshold of the following action potential. A complex and robust interplay between the activity of ion channels of the plasma membrane and the intracellular dynamics of Ca^2+^ underlies diastolic depolarization (Mangoni and Nargeot, 2008; Lakatta et al., 2010).

Among ion channels, hyperpolarization-activated cyclic nucleotide gated 4 (HCN4) channels underlying the “funny” current (*I*_f_) play an important role in SAN automaticity (DiFrancesco, 2010). In addition to *I*_f_, voltage-gated L- and T-type Ca^2+^ channels mediating L- and T-type Ca^2+^ currents (*I*_*CaL*_ and *I*_*CaT*_) contribute to the generation of SAN impulse. Indeed, they supply inward current at voltages spanning diastolic depolarization (Hagiwara et al., 1988; Verheijck et al., 1999; Mangoni et al., 2003, 2006b; Torrente et al., 2016). Together with type 2 ryanodine receptors (RyR2) of the sarcoplasmic reticulum (SR), *I*_f_ and *I_*CaL*_* mediate the positive chronotropic effect of catecholamines on SAN activity. In addition, the parasympathetic branch of the autonomic nervous system negatively regulates SAN pacemaker activity via two predominant pathways. First, vagally released acetylcholine activates muscarinic (M2) receptors to induce opening of G protein-gated inwardly rectifying K^+^ (GIRK) channels mediating the cardiac *I*_*KACh*_ current (Wickman et al., 1998). Second, activated M2 receptors promote down regulation of intracellular cAMP concentration, which reduces the amplitudes of *I*_f_ (DiFrancesco and Tromba, 1988a,b), *I*_*CaL*_ (Petit-Jacques et al., 1993), as well as intracellular RyR2-mediated Ca^2+^ release and cycling (Lyashkov et al., 2009; van Borren et al., 2010). The cardiac *I*_*KACh*_ current is mediated by heteromeric GIRK1/GIRK4 channel subunits (Krapivinsky et al., 1995). However, since GIRK1 subunits require GIRK4 to be properly targeted to the cell membrane, knockout of the *Girk4* gene induces genetic ablation of *I*_*KACh*_ in the heart (Wickman et al., 1998) and in the SAN (Mesirca et al., 2013).

In spite of its intrinsic robustness, several genetic- or disease- related factors may induce chronic slowing of pacemaker activity, a condition referred to as primary or secondary SAN (sinus node) dysfunction, respectively [SND, see Monfredi and Boyett (2015), Mesirca et al. (2020) for review]. SAN bradycardia, which can be associated with atrial tachyarrhythmia or atrioventricular block, characterizes SND (Brignole et al., 2013). Chronic symptomatic SND necessitates the implantation of an electronic pacemaker (Brignole et al., 2013). Intriguingly, there is now evidence that some veteran endurance athletes represent a subpopulation of acquired SND manifesting as bradyarrhythmia, and increased incidence of electronic pacemaker implantation (Northcote et al., 1989a,b) as well as AV node dysfunction (Stein et al., 2002) and atrial fibrillation (Andersen et al., 2013).

In rodent models of endurance training (D’Souza et al., 2014) and in human athletes (D’Souza et al., 2017), we have previously demonstrated an intrinsic slowing of SAN pacemaking attributable to training-induced transcriptional remodeling of key pacemaking ion channels. Specifically, in mice trained by swimming, we identified a role for transcriptional downregulation of the HCN4 channel (and a corresponding reduction in *I*_f_) in the development of training-induced bradycardia. As such, swim-training in rodents may be regarded as a model of secondary HCN4-mediated SND.

In previous work, we also showed that genetic ablation of *I*_*KACh*_ by knockout of *Girk4* rescued SAN bradycardia and prevented associated arrhythmias in mice expressing dominant negative non-conductive HCN4 subunits (Mesirca et al., 2014). Furthermore, we showed that ablation of *I*_*KACh*_ restores normal HR and rhythm in mice lacking L-type Ca_v_1.3 channels (*Ca_*v*_1.3^–/–^*) (Mesirca et al., 2016a; Bidaud et al., 2020). Finally, work on human SAN maintained *ex vivo* showed that pharmacologic block of *I*_*KACh*_ prevents failure of impulse generation and conduction induced by adenosine (Li et al., 2017). Taken together, these data have suggested that genetic or pharmacological targeting of *I*_*KACh*_ may constitute promising concepts to improve HR and rhythm in SND [see Mesirca et al. (2016b, 2020), for review].

However, evidence showing that genetic ablation of *I*_*KACh*_ can improve *in vivo* HR in secondary forms of bradycardia and SND are lacking. We thus investigated the consequences of *I*_*KACh*_ ablation on training-induced SAN bradycardia in mice and hypothesized that *I*_*KACh*_ channels are required for the development of training-induced bradycardia. We show that *I*_*KACh*_ ablation protects mice from training induced SAN bradycardia. *I*_*KACh*_ knockout blocked down-regulation of *I*_f_, *I*_*CaT*_ and *I_*CaL*_*, explaining protection of *Girk4*^–/–^ mice from training-induced bradycardia. Our study provides first evidence that genetic deletion of *I*_*KACh*_ can prevent bradycardia in an *in vivo* model of secondary SND.

## Materials and Methods

Wild-type (WT) and *Girk4*^–/–^ (Mesirca et al., 2013) mice were bred and maintained under the C57Bl/6J genetic background. The investigation conforms to the Guide for the Care and Use of Laboratory Animals published by the US National Institute of Health (NIH Publication No. 85–23, revised 1996) and European directives (2010/63/EU). The experimental procedure was approved by the Ethical committee of the University of Montpellier and the French Ministry of Agriculture (protocol n°: 2017010310594939). Animals were housed in individual cages with free access to food and water and were exposed to 12-h light/dark (light, 8:00 h to 20:00 h) in a thermostatically controlled room.

### Training Protocol

68 WT and 67 *Girk4*^–/–^ mice were assigned to a sedentary or trained group. Mice in the trained group first underwent a ramp-up period, in which the duration of swimming was increased in daily increments of 10 min, to finally reach 1 h. Mice in the trained group then underwent 1-h exercise swimming twice a day (morning session: 09:30–10:30, afternoon session 15:30–16:30) for 28 days, 7 days per week. Sedentary mice underwent 5-min swimming in the same period, to account for stress-related effects. The temperature of the water was set to 35°C (3 ppm Cl). After each session, mice were dried manually and then exposed to a warming red light source for 30 min.

### ECG Recordings in Conscious Mice and Heart Rate Variability Analysis

Mice undergoing telemetric ECG recordings were anesthetized with 2% isoflurane (Forene^®^, Abbott, United Kingdom). A midline incision was made on the back along the spine to insert a telemetric transmitter (ETA-F10, Data Sciences International) into a subcutaneous pocket. Paired wire electrodes were placed over the thorax (chest bipolar ECG lead) in DII derivation against the heart axis. Mice were left to recover for 14 days before ECG recordings. ECG signals were recorded using a telemetry receiver and an analog-to-digital conversion data acquisition system for display and analysis by Dataquest^TM^ A.R.T.^TM^ software (Data Sciences International). We recorded ECG for 12 h, before the ramp-up period (basal conditions) and daily (from 20:00 to 08:00 dark period) after ramp-up period until the 28th day of training. Heart rates (HR) were measured from ventricular RR intervals. ECG parameters were measured with ECG Auto 1.5.7 software (EMKA Technologies). HRV analysis was performed on telemetric ECGs by sampling four different 5-min periods of stable ECG segments (first 5-min period 22:55–23:00; second 5-min period 01:55–02:00; third 5-min period 04:55–05:00, and fourth 5-min period 07:55–08:00) at day 0 and at day 28 in WT and *Girk4*^–/–^ sedentary and trained animals. The standard deviation of intervals between two consecutive heart beats (SDNN), power spectral density (PSD) of HRV determined by Fast Fourier Transformation analysis (Welch Periodogram method), spectral frequency bands (low frequency spectra 0.15–1.5 Hz, high-frequency spectra 1.5–5 Hz and ratio between LF and HF values), percentage of successive intervals that differ by more than 6 ms (pNN6), standard deviation of instantaneous beat-to-beat interval variability (SD1) and continuous long-term R-R interval variability (SD2) provided by ellipse-fitting technique of the Poincaré scatter-gram obtained in each of the four 5 min period were averaged.

ECGs were also recorded from conscious restrained mice using the non-invasive ecgTUNNEL^®^ device (Emka Technologies). ECG signals were continuously recorded for 15 min using iOX Software v2.10.5.14 (Emka Technologies) and the heart rate was analyzed with ecgAUTO v3.3.5.12 (Emka Technologies). Each mouse underwent habituation to the setup for 10 min before data collection. ECG measurements started 40 min after intraperitoneal injection of saline or atropine (0.5 mg/kg, Aguettant) and propranolol (5 mg/kg, Sigma Aldrich) solution. This delay was considered as a good compromise between the absence of the artifact due to the stress of the injection and the measurement of the amplitude of the drug effect.

### Echocardiography and Arterial Pressure Recordings

Anesthetized mice (1–1.2% isoflurane) underwent transthoracic two-dimensional echocardiography. Images were obtained in parasternal long-axis view and short-axis view at the midpapillary muscle level. Cardiac morphology and function were assessed using high frequency, high-resolution echocardiographic system consisting of a VEVO ultrasound machine (2100) equipped with a 22–55 MHz bifrequencial transducer (VisualSonics B.V.), with continuous temperature and ECG monitoring.

Blood pressure was recorded using the CODA mouse tail-cuff system (Kent Scientific) in conscious restrained mice. Systolic and diastolic blood pressure were measured using volume pressure recording (VPR) to determine the tail blood volume (Daugherty et al., 2009) and recorded using the Coda 3.4 software (Kent Scientific). Pressure measurements started after 3 days of adaptation during which mice become accustomed to the holders and to tail cuff procedure. Recordings were always performed by the same investigator. Each session started with animals installed for 15 min in the holders placed on the warmed measurement platform. Following the 15-min habituation period, a set of 30 consecutive measurements was used for determining the blood pressure in each mouse.

### SAN Cell Isolation

Sinoatrial node cells were isolated as previously described (Mangoni and Nargeot, 2001). Briefly, SAN tissue strips were immersed into a “low-Ca^2+^” Tyrode’s solution containing 140 mM NaCl, 5.4 mM KCl, 0.5 mM MgCl_2_, 0.2 mM CaCl_2_, 1.2 mM KH_2_PO_4_, 50 mM taurine, 5.5 mM D-glucose, 1 mg/mL BSA, and 5 mM Hepes-NaOH (pH 6.9 with NaOH) for 5 min. The tissue was then transferred into a low-Ca^2+^ containing solution, washed 3 times and then transferred to a low-Ca^2+^ solution containing purified collagenase and protease mix (Liberase TM; 229 U/mL; Roche) and 1.9 U/ml elastase (Boehringer Mannheim). Digestion was carried out for 15–20 min at 36°C. SAN strips were then washed in a modified “Kraftbrühe” (KB) medium containing 70 mM L-glutamic acid, 20 mM KCl, 80 mM KOH, 10 mM KH_2_PO_4_, 10 mM taurine, 1 mg/ml BSA, and 10 mM Hepes-KOH (adjusted to pH 7.4 with KOH). Single cells were isolated from the tissue by manual agitation using a flame-forged Pasteur pipette in KB solution at 36°C. For recovering of pacemaker activity, Ca^2+^ was gradually reintroduced into the cell storage solution to a final concentration of 1.8 mM. Normal Tyrode solution containing 1 mg/ml BSA was added to the storage solution. Cells were then stored at room temperature until use.

### Patch-Clamp Recordings

We employed the whole-cell variation of the patch-clamp technique to investigate the effects of the training or sedentary regimen on *I_f_, I_*CaT*_* and *I*_*CaL*_ in SAN cells from wild-type or *Girk4*^–/–^ mice (Hamill et al., 1981). To this aim, cells were harvested in recording chambers (working volume 500 μl) allowing controlled unidirectional solution flow and mounted on the stage of an Olympus X71 inverted microscope. Cells were continuously perfused with normal Tyrode solution. Actions potentials and ionic currents were recorded using an Axon multiclamp patch-clamp 700B amplifier (Axon Instruments Inc.), grounded by an agar bridge filled with 150 mM KCl. Pacemaker activity was recorded by the perforated patch technique with escin (50 μM). Recording electrodes were pulled from borosilicate glass, using a DMZ-Universal Electrode Puller (Zeitz Instruments). For recording cell automaticity, as well as *I*_f_, we used an intracellular solution containing (mM): K^+^-aspartate, 130; NaCl, 10; ATP-Na^+^ salt, 2; creatine phosphate, 6.6; GTP-Mg^2+^, 0.1; CaCl_2_, 0.04 (pCa = 7); Hepes-KOH, 10 (adjusted to pH = 7.2 with KOH). Electrodes had a resistance of about 5 MΩ. The extracellular solution contained (in mM): NaCl, 140; KCl, 5.4; CaCl_2_, 1.8; MgCl_2_, 1; Hepes-NaOH, 5; and D-glucose, 5.5 (adjusted to pH = 7.4 with NaOH). Data was acquired with pClamp software (ver. 9, Axon Instruments Inc.). For recordings of *I*_*CaL*_, we used an extracellular solution containing (in mM): 135 tetraethylammonium chloride (TEA-Cl), 10 4-aminopyridine (4-AP), 1 MgCl_2_, 0.03 tetrodotoxin (TTX), 1 g/l glucose, 2 CaCl_2_, 10 Hepes (adjusted to pH = 7.2 with CsOH) (Mangoni et al., 2003). Electrodes had a resistance of about 3 MΩ when filled with an intracellular solution containing (in mM): 125 CsOH, 20 TEA-Cl, 1.2 CaCl_2_, 5 Mg-ATP, 0.1 Li_2_-GTP, 5 EGTA, and 10 HEPES (pH adjusted to 7.2 with aspartate). When recording *I*_f_ or *I*_*CaL*_, seal resistances were in the range of 2–5 GΩ.

The I_f_ steady-state activation curve was calculated as previously described (DiFrancesco and Mangoni, 1994). Briefly, an hyperpolarizing voltage ramp starting from an holding potential of −35 mv to −135 mV with 100 mV/80s rate was applied. The *I*_f_ activation curve was then calculated as the voltage dependence of probability of f-channels’ opening *P(V)*, by calculating the ratio between the current steady-state waveform and fully-activated I-V relationship (Mangoni and Nargeot, 2001). The fully activated I-V relationship was calculated by extrapolating the straight line passing through the point of zero current (fixed at −40 mV) and the point of maximal current (at −135 mV). Averaged I_f_ activation curve was then fitted according to a modified Boltzmann equation: *P(V)* = *1/[1* + *exp(V-V_1/2_)/k]*, where *P(V)* is the voltage dependency of the f-channels open probability, *V_1/2_* is the half-activation voltage and *k* is the slope factor. Analysis was performed using Prism software (v 8.4.1 GraphPad). Current densities and activation of *I*_*CaT*_ and *I*_*CaL*_ were calculated as described previously (Mangoni et al., 2003). Half-activation voltages were calculated by fitting current I-V curves by using the Boltzmann relation: *I/I_*max*_* = *g_*max*_(V − V_*rev*_){1* + *exp[(V_1/2_ − V)/k]}*, where *V*_*rev*_ is the current reversal potential, *V* is the membrane voltage, *I* is the peak current, *g*_*max*_ is the maximal conductance, *V_1/2_* is the voltage for half activation, and *k* is the slope factor.

### Numerical Modeling of Pacemaker Activity

Numerical simulations of pacemaker activity of mouse SAN cells were performed using a model that we developed previously (Christel et al., 2012). To simulate the effects of the training regimen on pacemaker activity, we entered the conductance of *I*_f_ and *I*_*CaL*_ recorded in sedentary and trained WT and *Girk4*^–/–^ mice. Equations to simulate *I*_f_ and *I*_*CaL*_ were the same as in Christel et al. (2012). Calculations were performed in the Jsim environment for integration of differential equations^1^. The integration step was set to 200 μs. Simulations were analyzed using the Graph Prism software (ver. 5.03).

### RNA Isolation and qPCR

Tissue biopsies were collected from the SAN of trained and sedentary mice approximately at the level of the main branch from the *crista terminalis*. Biopsies were frozen in liquid N_2_ and stored at −80°C until use. RNA was isolated using a Qiagen RNEasy kit following manufacturer’s instructions. For mRNA quantitation of HCN4, Ca^2+^ channel accessory subunits and miRs, cDNA was generated using the miScript II RT kit (Qiagen), using the HiFlex buffer option, to allow analysis of miRs and mRNAs in the same cDNA sample. The reaction mixture for mRNA comprised 1 μl of cDNA, 1 × Qiagen assay, 1 × SYBR Green Master Mix (Applied Biosystems) and DNase-free water. mRNA expression was calculated by the ΔCt method and normalization to the expression of *Tbp* which was determined as the optimal endogenous control (*Polr2a*, *Tbp* and *Ubc* were tested) using the algorithm geNorm (qBaseplus, version 2.0, Biogazelle). The miScript SYBR green PCR kit was used to measure miR expression. The reaction comprised 1 μl cDNA, 1 × miScript universal primer, 1 × primer assay and DNAse-free water.

Primers were purchased from Qiagen (formerly Exiqon, miR-10b-5p, 205637; miR-486-3p, 204107; miR423-5p, 205624; miR-676-3p, 205098; miR-181b-5p, 204530; Let-7e-5p, 205711; Let-7d-5p, and 204124). Primer set for mmu-miR-5099 was custom designed according to previously published sequences. miR expression was calculated by the ΔCt method with normalization to expression of *Snord65* and *Snord91* (geNorm-determined optimal reference gene combination, *Snord65* and *Snord91* and *Rnu1a1* tested). All samples were run in duplicate. mRNA expression of L-type Ca^2+^ channel subunits as well as GIRK1 and GIRK4 was carried out using custom-designed TaqMan Low Density Array microfluidic cards (Applied Biosystems, cat. no. 2549025; format 96A) as described in detail elsewhere (D’Souza et al., 2014). mRNA expression for these transcripts was calculated by the ΔCt method and normalized to the expression of *Tbp*.

### Western Blots

Snap frozen sinus node biopsies were homogenized with RIPA buffer (Sigma Aldrich) with protease inhibitors in FastPrep lysing matrix D ceramic bead (1.4 mm) 2 mL tubes (MPBio) using an MP FastPrep-24. Pierce BCA protein assays were used to estimate total protein concentration following which samples were denatured in 5× laemmli buffer and 6 M urea and heated to 37°C for 15 min. Proteins were separated using a 4–20% or 7.5% stain-free SDS-polyacrylamide gel electrophoresis (PAGE; Bio-Rad) system with Precision Plus Unstained Protein Standards, *Strep*-tagged recombinant (Bio-Rad) running at 110V for ∼70 min in SDS running buffer (25 mM Tris, 192 mM glycine, 0.1% SDS). Stain-free gels were imaged using ChemiDoc MP and proteins transferred to PVDF (polyvinyl difluoride) membranes using the *Trans*-Blot Turbo transfer system and buffers (Bio-Rad) at 25 V/1Amp for 30 min according to the manufacturer’s instructions. For Ca_v_1.2 the ethanol in the transfer buffer was reduced to 10%. Successful transfer was confirmed by imaging using the ChemiDoc MP and an image was obtained for total protein quantification. PVDF membranes were washed in Tris-buffered saline containing 0.1% v/v Tween 20 (TBS-Tween) and blocked in 4% BSA in TBS-Tween and incubated with primary antibodies in 2% BSA. Rabbit polyclonal anti-HCN4 (Alomone Labs, APC-052, Lot #APC052AN2802), anti-Ca_v_1.2 (ACC-003, Lot #ACC003AN6802), anti-GIRK1 (APC-005, Lot #APC005AN1125) and anti-GIRK4 (APC-027, Lot #APC027AN0725), were used at 1:200. anti-Ca_v_1.3 (Christel et al., 2012) was used at 1/2000. After washing, membranes were then incubated with horseradish peroxidase (HRP)-linked secondary antibody (HRP-linked anti-rabbit IgG, Cell Signaling, 1:3000) and Precision Protein StrepTactin-HRP conjugate (Bio-Rad, 1:5000) in milk-TBS-Tween. Unbound secondary antibody was removed by washing in TBS-Tween following which membranes were treated with Clarity Western ECL substrate (Bio-Rad) and imaged with a Bio-Rad ChemiDoc MP system. The chemiluminescent signal intensity was normalized to quantification of total protein, calculated and volume-adjusted using Image Lab 6.0 by selection of equivalent lane segments across the blot on the total protein image. All samples were run in duplicate. For HCN4, Ca_v_1.2 and Ca_v_1.3, wild-type and *Girk4*^–/–^ samples were run on separate gels and data given as% reduction from corresponding sedentary control (set as 100%).

### Data Analysis and Statistics

Data analysis and statistical assessing were performed using Prism 8.0 (GraphPad Software). Data are represented as mean ± SEM unless differently stated. Statistical tests used in each experiment are specified throughout the figure legends. In the text and in the legends, statistical significance was defined as *p* < 0.05. ^∗^*p* < 0.05, ^∗∗^*p* < 0.01, ^∗∗∗^*p* < 0.001, and ^****^p < 0.0001.

## Results

### Genetic Ablation of *I*_*KACh*_ Prevented Training-Induced SAN Bradycardia

**FIGURE 1:**
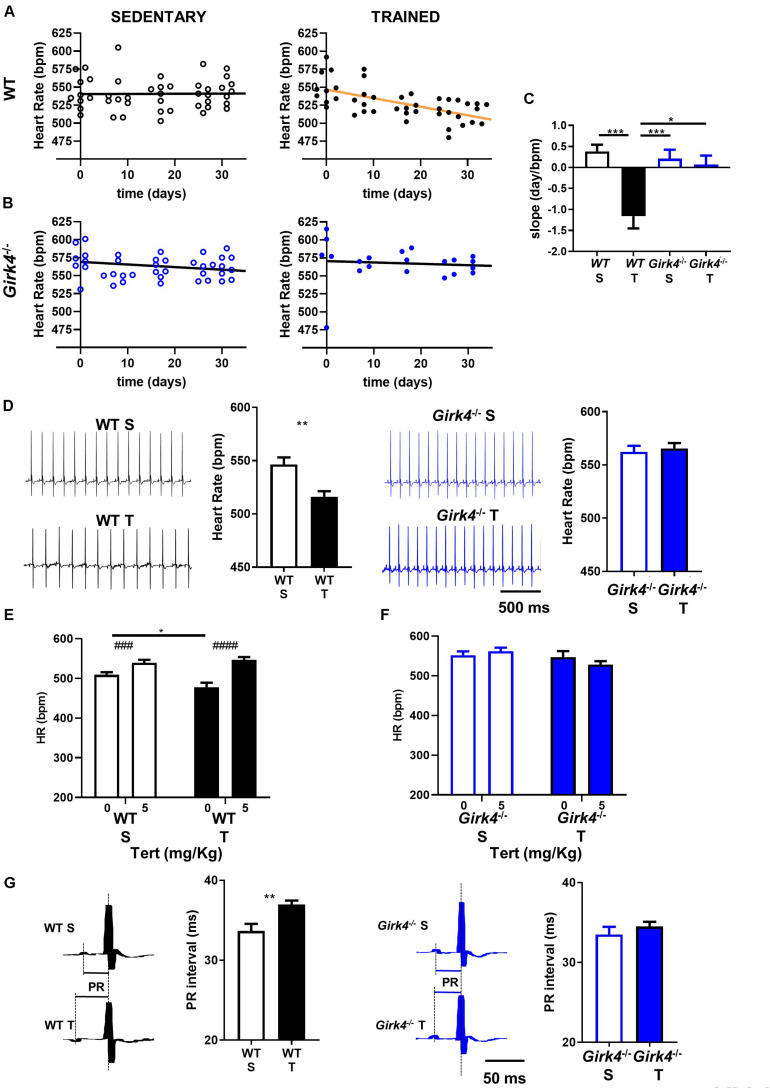
Heart rate recorded in sedentary (left panel) and trained (right panel) WT **(A)** and *Girk4*^–/–^ mice **(B)** at different days during 5 min sham-training (WT and *Girk4*^–/–^ sedentary, see methods) and training (WT and *Girk4*^–/–^ trained) protocol. **(C)** Histogram of the average values of the slopes of the regression line between time and heart rate. Statistics: one-way analysis of variance followed by Tukey’s multiple comparisons test. **(D)** Representative examples of ECG traces and averaged heart rate recorded at day 28 from sedentary (top) and trained (bottom) WT (left panel) and *Girk4*^–/–^ mice (right panel). Statistics: unpaired Student’s *t*-test. Tertiapin-Q (Tert, 5 mg/kg) effect in WT **(E)** and *Girk4*^–/–^ **(F)** sedentary (empty bar) and trained (filled bars) mice. Statistics: two-way analysis of variance followed by Sidak multiple comparisons test. **(G)** Close-up of ECG traces and averaged PR interval recorded at day 28 from sedentary (top) and trained (bottom) WT (left panel) and *Girk4*^–/–^ mice (right panel). Statistics: unpaired Student’s *t*-test. *p < 0.05, **p < 0.01, ***p < 0.001, ^###^*p* < 0.001, ^####^*p* < 0.0001. Error bars indicate s.e.m. WTS: WT sedentary; WTT: WT trained; *Girk4*^–/–^ S: *Girk4*^–/–^ sedentary and *Girk4*^–/–^ T: *Girk4*^–/–^ trained.

We compared the HR of mice assigned to the trained group to that of the sedentary group (Figure 1). The HR of trained WT mice decreased with training progression and was significantly reduced by day 17 (550 ± 7 bpm vs 522 ± 4 bpm, *p* < 0.05, day 0 and day 17, respectively, Figure 1A). In contrast, the HR rate of trained *Girk4*^–/–^ mice remained stable throughout the training regimen (Figure 1B). As expected, the HR of sedentary WT or *Girk4*^–/–^ mice remained unaltered (Figures 1A,B). As such, a significant change in the slope of the regression line between HR and days of regimen only in the group of trained WT mice (Figure 1C). At swimming cessation (day 28), the HR of trained WT mice was significantly lower that of sedentary counterparts. In contrast, we failed to observe a statistically significant difference in HR between trained and sedentary *Girk4*^–/–^ mice (Figure 1D).

The effect of tertiapine-Q on HR was investigated in sedated mice using echocardiography (see Methods). Sedated WT mice presented with slightly reduced basal heart rate (Figure 1E) in comparison to conscious animals (Figure 1D). Administration of tertiapin-Q increased the HR of sedentary mice, in line with inhibition of tonic regulation of HR by *I*_*KACh*_, as we reported previously (Mesirca et al., 2016a). In addition, tertiapin-Q increased the HR of trained WT (Figure 1E), but not *Girk4*^–/–^ mice (Figure 1F). The HR of sedentary and trained WT mice after administration of 5 mg/Kg tertiapin-Q was similar, showing that pharmacologic inhibition of *I*_*KACh*_ compensated for the decrease in HR induced by the training regimen.

Absence of significant training-induced bradycardia in *Girk4*^–/–^ mice could not be attributed to reduced training activity of mutants compared to WT animals, because the difference in body weight between sedentary and trained mice at the end of training regimen was similar in both strains- (Supplementary Figure 1).

Besides HR, the training regimen also prolonged the atrioventricular conduction (PR) interval in WT mice (33.6 ± 0.9 ms vs 37.0 ± 0.5 ms, *p* < 0.01). In contrast, the PR intervals of *Girk4*^–/–^ mice did not change significantly upon training (Figure 1G). We did not detect statistically significant differences in QRS, QT and QTc intervals of the ECG waveform between WT and *Girk4*^–/–^ mice, either in the sedentary condition or after 28 days of training (Supplementary Table 1). *In vivo* echocardiographic imaging of sedentary and trained WT and *Girk4*^–/–^ mice showed slowing of HR in trained WT animals to be associated with hallmarks of ventricular hypertrophy (Supplementary Figure 2). Particularly, the training regimen significantly increased the left ventricular mass diameter and volume, as well as wall thickness. These changes were accompanied by an increase in ventricular stroke volume and a decrease in ejection fraction. Remarkably, the training regimen did not affect ventricular morphology, stroke volume or ejection fraction in *Girk4*^–/–^ mice (Supplementary Figure 2). Finally, no changes in arterial systolic or diastolic pressure in WT and *Girk4*^–/–^ mice were noted (Supplementary Figure 3). Cumulatively, these data demonstrate, for the first time, the requirement of *I*_*KACh*_ in the development of training-induced sinus bradycardia as well as canonical structural and functional remodeling characteristics commonly referred to as the ‘athlete’s heart.’

**FIGURE 2:**
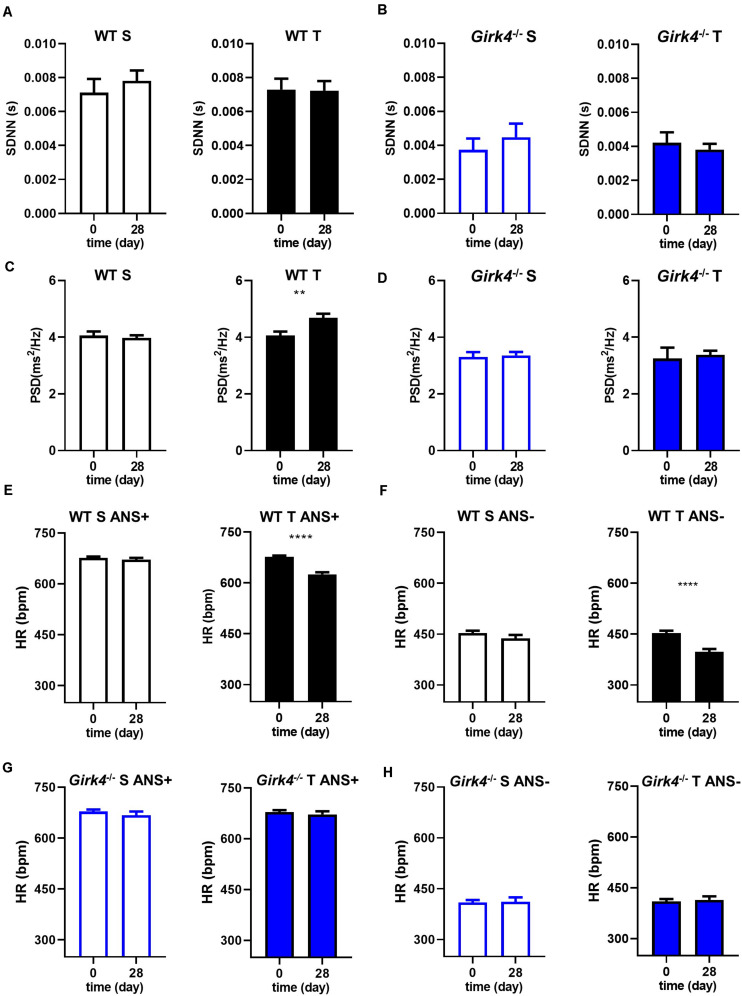
Standard deviation of intervals between two consecutive heart beats (SDNN) calculated in 5-min stable ECG periods at day 0 and at day 28 in WT **(A)** and *Girk4*^–/–^ **(B)** sedentary (open bars) and trained (filled bars) animals. Power spectral density (PSD) of heart rate variability determined by Fast Fourier Transformation analysis of 5-min of stable ECG segments at day 0 and at day 28 in WT **(C)** and *Girk4*^–/–^ **(D)** sedentary (open bars) and trained (filled bars) animals. Heart rate (non-invasive ECG recordings) measured in sedentary (open bars, left panel) and trained (filled bars, right panel) WT mice before (day 0) and after (day 28) training period in control condition (ANS +, **E**) or following intraperitoneal injection of atropine (0.5 mg/kg) and propranolol (5 mg/kg) to inhibit the input of the autonomic nervous system (ANS-, **F**). **(G,H)** same as **(E,F)** but in *Girk4*^–/–^ animals. Statistics: unpaired Student’s *t*-test. **p < 0.01, ****p < 0.0001. Error bars indicate s.e.m.

Mechanisms underlying the prevention of training-induced bradycardia in *Girk4*^–/–^ mice were investigated. The heart rate adaptation to training is widely attributed to high parasympathetic (vagus) activity and therefore we studied the impact of training on heart rate variability (HRV, a surrogate measure of autonomic activity) in WT and *Girk4*^–/–^ mice (Figures 2A–D). Genetic ablation of *I*_*KACh*_ reduced the standard deviation of the RR interval (SDNN) in comparison to WT counterparts (Figures 2A,B), as previously reported for this mouse strain (Wickman et al., 1998; Mesirca et al., 2013). However, the measured SDNN was similar at day 0 and day 28 in WT and in *Girk4*^–/–^ mice (Figures 2A,B), suggesting that the training regimen did not affect the autonomic innervation of the SAN. Consistent with this hypothesis, training did not affect the integral of the low-frequency (LF) or high-frequency (HF) fractions of the HRV spectrum in WT or *Girk4*^–/–^ mice (Supplementary Figures 4A,B). Furthermore, the LF/HF ratio did not differ between WT and *Girk4*^–/–^ mice and was unaffected by training (Supplementary Figure 4C). The training regimen significantly augment the power spectral density (PSD) in WT but not in *Girk4*^–/–^ mice (Figures 2C,D). Finally, training did not affect other HRV parameters (pNN6, SD1 and SD2, Supplementary Figures 5A–C). We did not see significant sex differences in the HR, PR interval and in the power spectral density of heart rate variability neither in WT nor in *Girk4*^–/–^ animals under basal conditions (Supplementary Dataset 1). We then tested if decrease in HR observed in WT mice following the training regimen was maintained after pharmacologic inhibition of autonomic nervous system input. To this aim, we compared the HR (recorded non-invasively by TUNNEL-ECG see methods) of sedentary and trained WT and *Girk4*^–/–^ mice under control conditions or following concomitant injection of atropine (0.5 mg/Kg) and propranolol (5 mg/Kg). Concomitant injection of atropine and propranolol decreased HR in both sedentary and trained WT and *Girk4*^–/–^ mice (Figures 2E–H). However, the HR of trained WT mice was significantly lower than that recorded in sedentary counterparts (Figures 2E,F), which indicates that training induced slowing of intrinsic SAN pacemaker activity, as previously shown (D’Souza et al., 2014). In contrast, the training regimen did not significantly change the intrinsic SAN rate of *Girk4*^–/–^ mice (Figures 2G,H). Taken together, our results show that the training regimen induced SAN bradycardia, increased the AV conduction interval and reduced the intrinsic SAN rate in WT, but not in *Girk4*^–/–^ mice. In addition, our data show that training induces slowing of intrinsic SAN pacemaking at the end of training regimen, in the absence of a change in the sympathovagal balance.

### Genetic Ablation of *I*_*KACh*_ Abolished Training-Induced Reduction in Spontaneous Firing of SAN Cells

**FIGURE 3:**
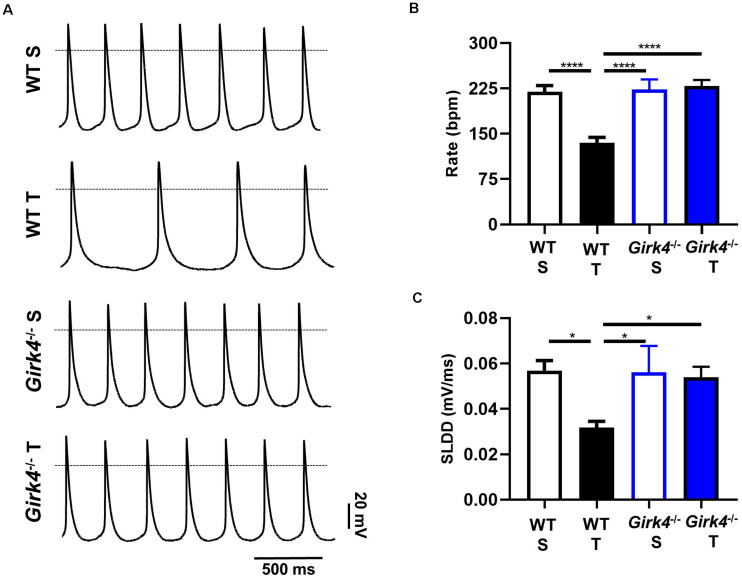
Action potential recordings of SAN myocytes isolated from WT sedentary (WT S), WT trained (WT T), *Girk4*^–/–^ sedentary (*Girk4*^–/–^ S) and *Girk4*^–/–^ trained (*Girk4*^–/–^ T) in Tyrode’s solution at the end of training, or sham-training protocol (day 28) **(A)**. The dotted line indicates the 0 mV. Rate of spontaneous action potentials **(B)** and slope of the linear part of the diastolic depolarization (SLDD, **C**) recorded at day 28 in SAN myocytes in Tyrode’s solution (*n* = 6 WT S, *n* = 10 WT T, *n* = 7 *Girk4*^–/–^ S and *n* = 8 *Girk4*^–/–^ T). Statistics: one-way analysis of variance followed by Tukey’s multiple comparisons test. *p < 0.05, ****p < 0.0001. Error bars indicate s.e.m.

Since the training regimen induced slowing of intrinsic SAN pacemaker activity in trained WT but not in *Girk4*^–/–^ mice, we recorded spontaneous action potentials from isolated pacemaker cells (Figure 3A). Consistent with recordings of resting HR *in vivo* under control conditions or following inhibition of autonomic nervous system input, the averaged spontaneous beating rate of SAN cells from trained WT mice was significantly lower than the rate of cells from the SAN of sedentary WT mice (135 ± 9 vs 220 ± 10 bpm, *p* < 0.0001, i.e., 39%, Figure 3B). The rate of spontaneous action potentials of *Girk4*^–/–^ SAN cells did not differ between the sedentary and trained group (223 ± 17 vs 229 ± 10 bpm, Figure 3B). Consistent with the effect on the rate of spontaneous action potentials, the slope of the linear part of the diastolic depolarization (SLDD) was significantly reduced by the training regimen (44%) in WT but not in *Girk4*^–/–^ mice (Figure 3C). The training regimen also significantly prolonged the action potential duration in WT but not in *Girk4*^–/–^ mice (Supplementary Table 2). No significant difference was observed in the rate of spontaneous action potentials of SAN cells obtained from sedentary WT and *Girk4*^–/–^ mice (Figure 3B). Finally, in sedentary or trained WT and *Girk4*^–/–^ mice, we did not record significant differences in the maximum diastolic potential, action potential threshold, slope of the exponential fraction of diastolic depolarization, action potential upstroke, and action potential amplitude (Supplementary Table 2). In conclusion, our data show that the training regimen slowed the HR by reducing the intrinsic spontaneous activity of SAN pacemaker cells and that genetic ablation of *I*_*KACh*_ prevented the reduction in spontaneous pacemaker activity.

### Genetic Ablation of *I*_*KACh*_ Prevented Training-Induced Down Regulation of *I*_f_, *I*_*CaT*_ and *I_*CaL*_* in SAN Cells

**FIGURE 4:**
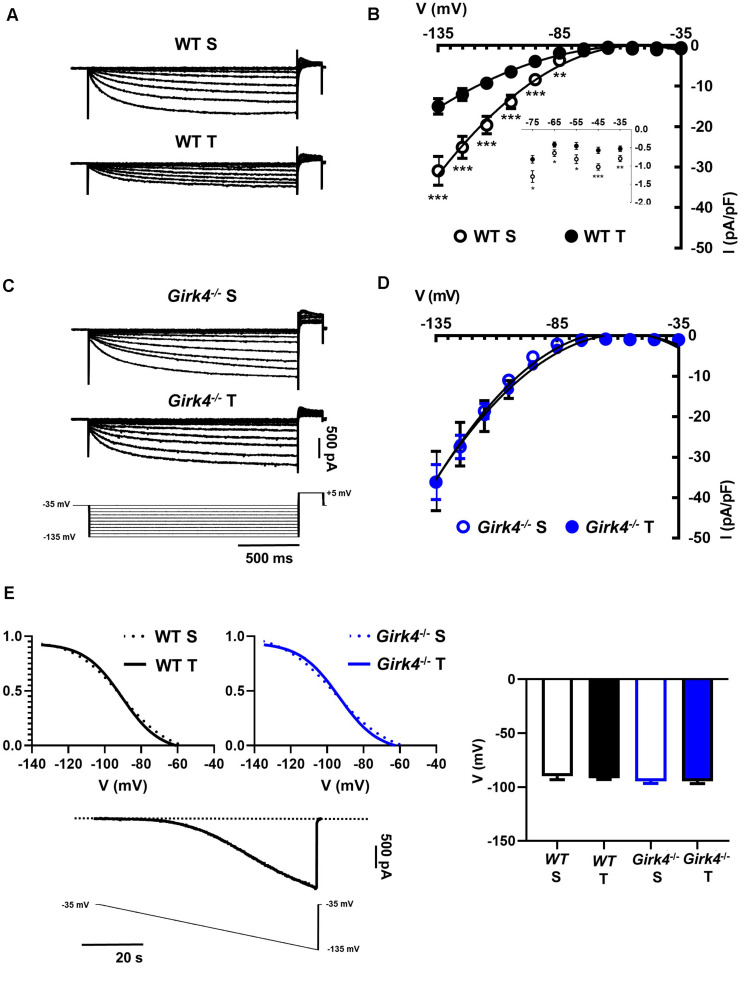
Representative traces of *I*_f_ recordings **(A)** and averaged current-to-voltage (I-V) curve **(B)** in sedentary (open black circles, *n* = 15) and trained (filled black circles, *n* = 23) WT SAN myocytes. *I*_f_ recordings **(C)** and I-V curve **(D)** in SAN myocytes from sedentary (open blue circles, *n* = 12) and trained (filled blue circles, *n* = 18) isolated *Girk4*^–/–^ SAN pacemaker cells. The voltage-clamp protocol used for all the recordings is shown at the bottom of panel **(C)**. Statistical significance was tested at each voltage using the unpaired Student’s *t*-test. *p < 0.05, **p < 0.01, ***p < 0.001. (**E**, left panel) Steady state *I*_f_ activation curves in isolated SAN cells from sedentary (dotted line) and trained (continuous line), WT (black) and *Girk4*^–/–^ (blue) mice. Representative ramp current trace recorded in a WT sedentary SAN cell and corresponding voltage protocol are shown under the curves. (**E**, right panel) Histograms representing averaged half-activation voltages (V_1/2_) values for *I*_f_ recorded in SAN cells from WT (black bars) and *Girk4*^–/–^ (blue bars) sedentary (open bars) and trained (filled bars) animals. Data have been collected at day 28 (end of training, or sham-training, protocol). Statistics: one-way analysis of variance. Error bars indicate s.e.m.. WT S: WT sedentary; WT T: WT trained; *Girk4*^–/–^ S: *Girk4*^–/–^ sedentary and *Girk4*^–/–^ T: *Girk4*^–/–^ trained.

**FIGURE 5:**
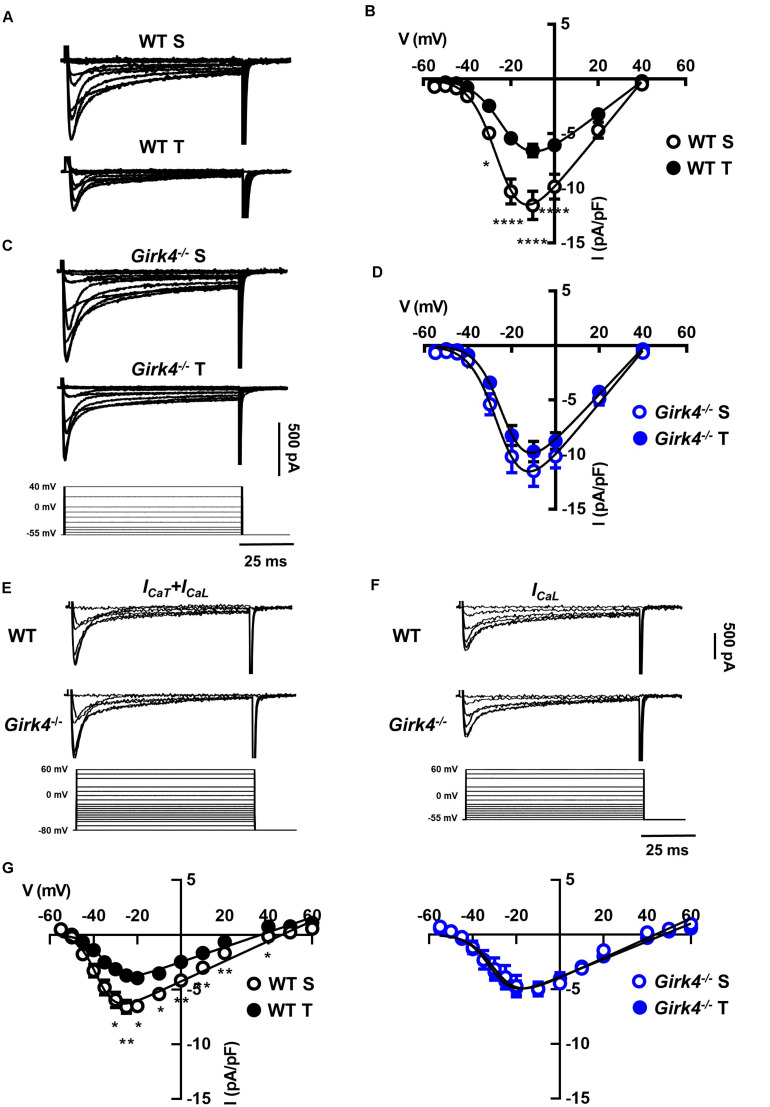
Representative Ca^2+^ current traces of the L-type Ca^2+^ current (*I*_CaL_) measured from a holding potential (HP) of −55 mV **(A)** and current-to-voltage (I-V) relationships **(B)** from sedentary (open black circles, *n* = 14) and trained (filled black circles, *n* = 13) WT SAN myocytes. Traces of *I*_CaL_ **(C)** and I-V curves **(D)** from sedentary (open blue circles, *n* = 15) compared with trained (filled blue circles, *n* = 22) *Girk4*^–/–^ SAN myocytes. Data, collected at day 28 were fitted with a modified Boltzmann equation. Voltage protocol is shown in panel C (bottom). **(E)** Sample traces of *I*_Ca_ (*I*_CaT_ + *I*_CaL_) recorded from a HP of −80 mV in WT and *Girk4*^–/–^SAN myocytes. **(F)** Sample *I*_Ca_ traces for same myocytes as in **(E)**, but after switching to HP = −55 to inactivate *I*_CaT_. **(G)** Net peak *I*_CaT_ I-V curves measured following subtraction of traces recorded from HP = −55 mV from traces obtained at HP = −80 mV in WT (left panel) and in *Girk4*^–/–^ (right panel) SAN myocytes from sedentary and trained mice. Statistical significance was tested at each voltage using the unpaired Student’s *t*-test. *p < 0.05, **p < 0.01, ****p < 0.0001. Error bars indicate s.e.m. WT S: WT sedentary; WT T: WT trained; *Girk4*^–/–^ S: *Girk4*^–/–^ sedentary, and *Girk4*^–/–^ T: *Girk4*^–/–^ trained.

Previous work showed that SAN bradycardia induced by training is due to downregulation of *I*_f_ (D’Souza et al., 2014). We thus compared the density of *I*_f_ in SAN cells from trained and sedentary WT and *Girk4*^–/–^ mice (Figure 4). *I*_f_ density in SAN cells from trained WT mice was significantly lower than in cells from sedentary mice (Figures 4A,B). *I*_f_ was reduced by about 40% at voltages spanning the range of diastolic depolarization (Figure 4B, inset). In contrast, the training regimen did not significantly affect *I*_f_ density in *Girk4*^–/–^ SAN cells (Figures 4C,D). *I*_f_ half-activation voltage was unaffected by the training regimen and was similar in both genotypes (Figure 4E). We then measured the density of *I*_*CaL*_ and *I*_*CaT*_ in SAN cells isolated from trained and sedentary WT and *Girk4*^–/–^ mice (Figure 5). The training regimen significantly reduced peak *I*_*CaL*_ density in WT but not in *Girk4*^–/–^ mice (Figures 5A–D). In addition, the training regimen shifted the current half-activation to more positive voltages (Supplementary Figure 6A). We then measured *I*_*CaT*_ in sedentary and trained WT and *Girk4*^–/–^ (Figures 5E–G). We separated *I*_*CaT*_ from *I*_*CaL*_ by subtracting traces recorded by stepping from a holding potential of −80 mV from those recorded from a holding potential of −55 mV, which completely inactivates *I*_*CaT*_ (Figures 5E,F; Mangoni et al., 2006b). The training regimen significantly reduced *I*_*CaT*_ density in WT but not in *Girk4*^–/–^ SAN cells (Figures 5G,H), leaving unaffected the current half-activation voltage (Supplementary Figure 6B). We did not find significant differences in densities of *I*_*CaL*_ and *I*_*CaT*_ between sedentary WT and *Girk4*^–/–^ mice (Figures 5B,D,G). Finally, the density of *I*_*KACh*_ was similar in sedentary and trained WT mice (Supplementary Figure 7), which indicated that HR slowing in trained WT animals could not be attributed to alterations in this current.

### Genetic Ablation of *I*_*KACh*_ Suppressed Training-Induced Molecular Remodeling of the SAN

**FIGURE 6:**
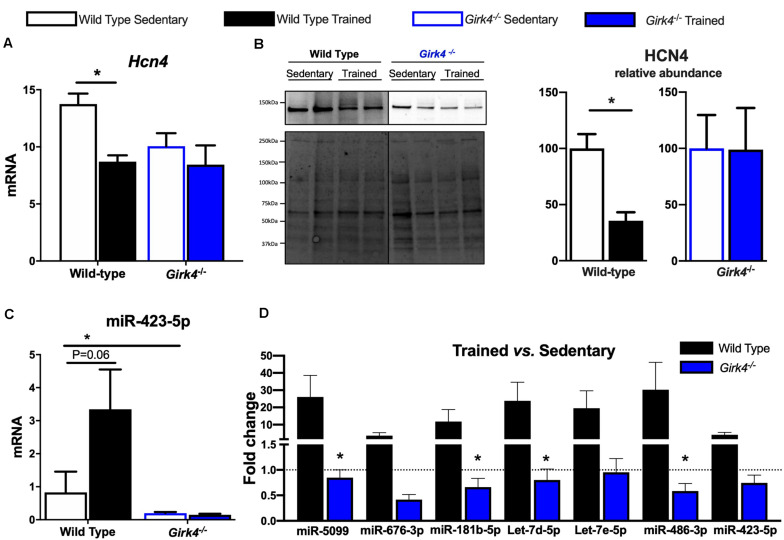
Expression of HCN4 mRNA **(A)** normalized to expression of *Tbp* in SAN biopsies of from WT sedentary (*n* = 8), WT trained (*n* = 8), *Girk4*^–/–^ sedentary (*n* = 7), and *Girk4*^–/–^ trained (*n* = 6) mice. Statistics: two-way analysis of variance with Sidak’s multiple comparisons test. Representative HCN4 western blot (**B**, left panel) with corresponding stain-free total protein gel used for quantification (lower left panel). (**B**, right panel) Protein expression determined by western blot in individual SAN biopsies isolated from WT sedentary (*n* = 4), WT trained (*n* = 4) sedentary *Girk4*^–/–^ mice (*n* = 5) and trained *Girk4*^–/–^mice (*n* = 4). Statistics: Students *t*-test. SAN Expression of miR-423-5p **(C)** in WT sedentary (*n* = 8), WT trained (*n* = 7) sedentary *Girk4*^–/–^ mice (*n* = 8) and trained *Girk4*^–/–^mice (*n* = 7). **(D)** Fold change in expression of selected miRs in the SAN of WT trained (*n* = 8) and *Girk4*^–/–^ trained mice (*n* = 7) relative to respective WT sedentary (*n* = 8) and *Girk4*^–/–^ sedentary mice (*n* = 7). Statistics: one-way analysis of variance with Tukey’s multiple comparisons test. *p < 0.05. miR expression in **(C,D)** was normalized to expression of Snord61 and Snord95.

We investigated the molecular underpinnings of the observed training-induced reduction in the density of the aforementioned inward currents and its abolition on *I*_*KACh*_ ablation. Consistent with previous findings (D’Souza et al., 2014, 2017), we confirmed that the training-induced reduction in *I*_f_ seen in WT mice was concomitant with a significant reduction in *Hcn4* (Figure 6A, *p* < 0.05) that translated into a reduced expression of HCN4 protein as determined by western blot (Figure 6B, *p* < 0.05). A representative western blot is shown in Figure 6B-left (top panel) and corresponding stain free total-protein gel used for quantification is given in the lower panel. In line with an unchanged *I*_f_ density in trained *Girk4*^–/–^mice (Figures 4C,D), training-induced HCN4 modulation was not detectable in *Girk4*^–/–^mice at either transcript (Figure 6A) or protein level (Figure 6B). From a separate set of experiments, it was determined that unlike mRNA, HCN4 protein levels did not vary between sedentary WT and *Girk4*^–/–^mice (Supplementary Figure 8).

We previously demonstrated that induction of a repressive miR signature (with specific emphasis on miR-423-5p, D’Souza et al., 2017) is a candidate mechanism for *Hcn4* downregulation in the trained WT SAN. Therefore, the consequences of *I*_*KACh*_ ablation for selected (previously investigated D’Souza et al., 2017) miRs were tested. Strikingly, induction of *Hcn4*-repressor miRs including miR-423-5p (Figure 6C) observed in trained WT mice was largely abrogated by *Girk4* silencing (Figure 6D). An intriguing baseline reduction in the expression levels of *Hcn4*, miR-423-5p, miR-676-3p and miR-181b-5p in sedentary WT vs sedentary *Girk4*^–/–^ mice was also noted but not investigated further (data not shown). Nevertheless, our findings highlight a new and complex association between *I*_*KACh*_ ablation and miR-mediated transcriptional regulation of *Hcn4* in the mouse SAN.

**FIGURE 7:**
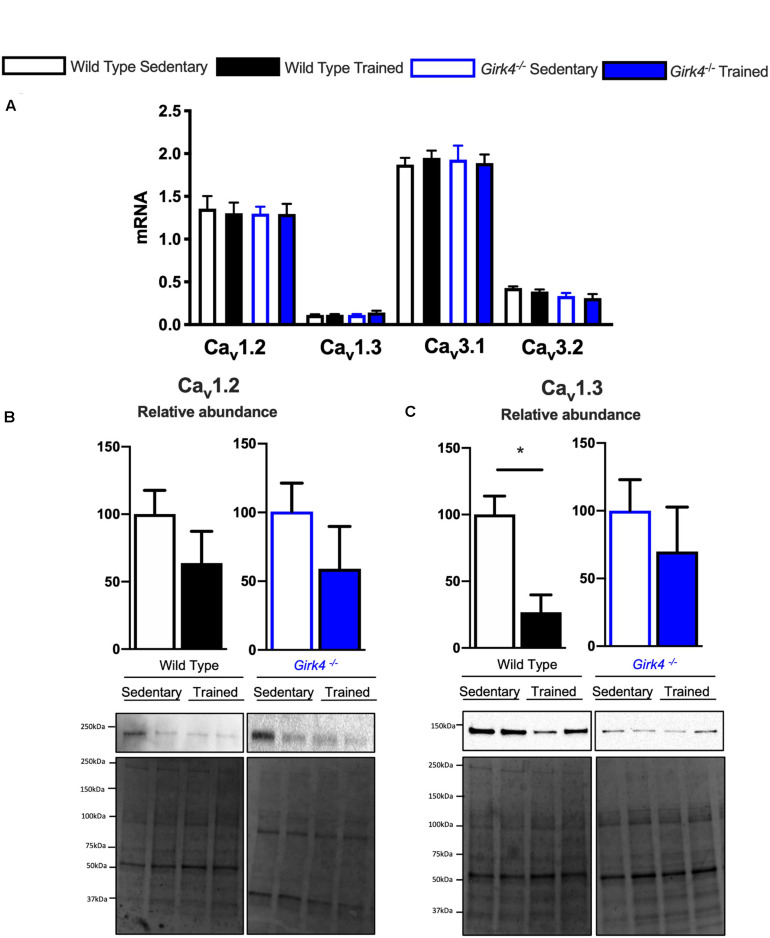
mRNA expression of L-type calcium channel subunits Ca_v_1.2, Ca_v_1.3, Ca_v_3.1, and Ca_v_3.2 **(A)** (normalized to expression of Tbp) in SAN biopsies from from WT sedentary (*n* = 10), WT trained (*n* = 10), *Girk4*^–/–^ sedentary (*n* = 9) and *Girk4*^–/–^ trained (*n* = 10) mice. Protein expression determined by western blot using antibodies directed against Ca_v_1.2 **(B)** and Ca_v_1.3 **(C)** in individual sinus node biopsies isolated from sedentary WT (*n* = 6), trained WT (*n* = 4) sedentary *Girk4*^–/–^ mice (*n* = 6) and trained *Girk4*^–/–^mice (*n* = 6). Representative western blots with corresponding stain-free total protein blot used for quantification shown in lower panel. Statistics: Students *t*-test. *p < 0.05.

Next, we assessed whether a similar transcriptional control mechanism extended to observed changes in *I*_*CaL*_ and *I*_*CaT*_. In contrast to *Hcn4*, mRNA expression of the α subunits of voltage-gated T- and L-type Ca^2+^ channels, Ca_v_1.2, Ca_v_1.3, Ca_v_3.1, and Ca_v_3.2 (Figure 7A) in trained and sedentary WT and *Girk4*^–/–^ mice were unaltered by training or *Girk4* silencing. Furthermore, expression of L-type Ca^2+^ channel α_2_δ and β subunit isoforms also remained unchanged (Supplementary Figure 9). At the protein level, western blotting demonstrated a significant training-induced reduction in the SAN expression levels of Ca_v_1.3 (Figure 7C, *p* < 0.05), but not of Ca_v_1.2 (Figure 7B) in WT mice. Consistent with Figures 5C,D,G (right panel) the training regimen did not significantly alter the expression levels of either ion channel subunit in the *Girk4*^–/–^ SAN, although there was a trend toward reduction in both cases. Commercially sourced antibodies against *I*_CaT_ subunits Ca_v_3.1 and Ca_v_3.2 could not be validated. In sum, the available data from these pilot molecular investigations indicate a potential role for post-transcriptional and/or post-translational modifications in explaining the reduction of the L- type Ca^2+^ current in the trained WT SAN.

Finally, and in keeping with the finding that *I*_*KACh*_ density was unaffected by training (Supplementary Figure 7), there were no detectable training-induced changes to GIRK1 or GIRK4 at transcript or protein levels (Supplementary Figure 10) in WT or *Girk4*^–/–^mice.

### Numerical Modeling of Training-Induced Effects on SAN Pacemaking

**FIGURE 8:**
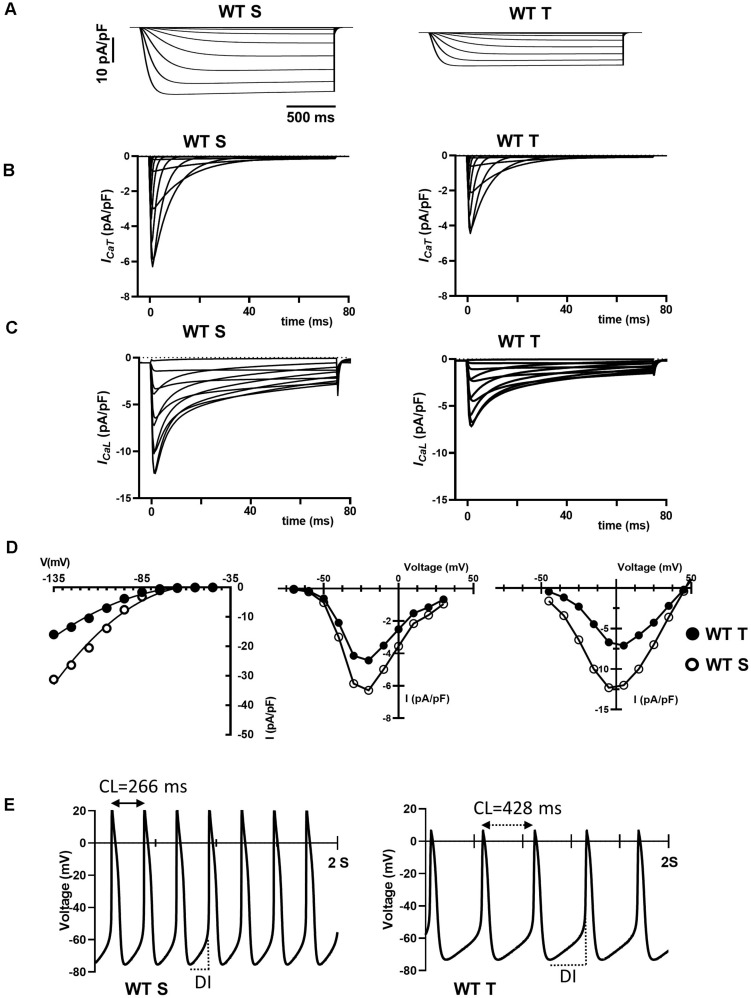
Numerical simulation of current traces for *I*_f_ **(A)**, *I*_*CaT*_ **(B)**, and *I*_*CaL*_ **(C)** calculated using current density and activation parameters recorded in WT sedentary (WT S), WT trained (WT T), *Girk4*^–/–^ sedentary (*Girk4*^–/–^ S) and *Girk4*^–/–^ trained (*Girk4*^–/–^ T) at day 28 of experimental protocol. **(D)** Corresponding predicted I-V curves of *I*_f_ (left panel), *I*_*CaT*_ (central panel) and *I*_*CaL*_ (right panel), calculated at the peak of current density from simulations in **(A–C)**. In I-V curves, open circles show predicted current densities of WT sedentary SAN cells and black circles densities of WT trained SAN cells. **(E)** Comparison between predicted pacemaker activities simulated for control (sedentary) condition (left panel), or including training-dependent changes of *I*_f_, *I*_*CaT*_ and *I*_*CaL*_. Abbreviations: CL, pacemaker activity cycle length; DI, diastolic interval.

The training regimen in WT mice affected *I_f_, I*_*CaT*_ and *I*_*CaL*_ (Figures 4, 5). Dissection of the contribution of these currents to training-induced decrease in automaticity of SAN cells, either individually or in combination, would be difficult to achieve using pharmacologic agents, as specific Ca_*v*_1.3 inhibitor or gating modifiers are yet to be identified. We thus attempted to predict the impact of training-induced regulation of these currents using a numerical model of mouse SAN cell automaticity that we developed previously (Christel et al., 2012; Figures 8A–E). Our model of pacemaker activity includes both L-type Ca_v_1.3 and Ca_v_1.2 isoform to calculate total *I*_*CaL*_, as the sum of Ca_v_1.3-mediated and Ca_v_1.2-mediated *I*_*CaL*_, respectively (Christel et al., 2012). When values for *I_f_, I*_*CaT*_ and *I*_*CaL*_ measured in sedentary WT SAN cells were used for calculations, the model generated basal pacemaking of 226 bpm, which compares to what recorded in native sedentary WT SAN cells (220 bpm, Figure 3B). To simulate the effects of training-induced reduction in *I*_f_ and *I*_*CaT*_ magnitudes in WT SAN cells, we used corresponding values of averaged densities and activation measured experimentally (Figures 4, 5 and Supplementary Figure 6). Because we observed decrease in protein expression of Ca_v_1.3 but not Ca_v_1.2 (Figure 7), we attributed the change in *I*_*CaL*_ magnitude and shift of the voltage for half activation to Ca_v_1.3-mediated *I*_*CaL*_. When all changes in *I_f_, I*_*CaT*_ and *I*_*CaL*_ are included the model predicted a 38% slowing of predicted pacemaker activity, which compares to 39% observed in native trained WT SAN cells (from 226 to 140 bpm, Figure 8E). We then calculated the predicted relative effect of changes in each current individually (Supplementary Figure 11). The model predicted a 15% slowing of automaticity when only the training-induced reduction in *I*_f_ density was included in the simulation. Similarly, computed automaticity predicted 13% slowing of pacemaking when only the training-induced change in *I*_*CaT*_ magnitude was included in calculations. A very limited (1%) prolongation of the computed pacemaker cycle length was obtained when changes in *I*_*CaL*_ magnitude and in voltage for half activation were included. Change in total *I*_*CaL*_ also reduced the predicted action potential amplitude. However, analysis of diastolic depolarization phase showed that the diastolic interval (DI) was prolonged by 11%, by concomitant reduction in *I*_*CaL*_ magnitude and the + 4 mV positive shift in the current voltage for half activation, which indicates slowing of the computed pacemaker mechanism. In contrast, predicted action potential duration was reduced by training-induced changes in *I*_*CaL*_ explaining in part the lack of significant slowing of pacemaker cycle length despite the effect on diastolic interval. In contrast, when changes in *I*_*CaL*_ magnitude corresponding to values recorded in sedentary and trained *Girk4*^–/–^ cells were included in the model, no significant training-induced slowing in the rate of pacemaker activity was predicted (Supplementary Figure 12), in line with experimental data. Taken together, these results show that genetic ablation of *I*_*KACh*_ prevents training-induced remodeling of ionic currents in SAN cells and that remodeling of *I*_f_, *I*_*CaT*_ and *I*_*CaL*_ contribute to this effect.

## Discussion

### Impact of the Study

This is the first demonstration that that genetic ablation of *I*_*KACh*_ prevents sinus bradycardia, slowing of atrioventricular conduction and development of ventricular hypertrophy in a murine model of athletic training. The novel findings of this study accumulate along three lines: First, we report that endurance exercise downregulates not only *I*_f_ but also *I*_*CaT*_ and *I*_*CaL*_ and the *combination* of these changes predicts HR reduction *in silico.* Second, we show that training-induced remodeling of *I*_f_, *I*_*CaT*_ and *I*_*CaL*_ is suppressed by *I*_*KACh*_ ablation, explained in part by: (i) a previously unsuspected transcriptional interaction with HCN4 and its repressor miRs and (ii) likely post-transcriptional regulation of Ca_v_1.3. Finally, we also demonstrate that acute *I*_*KACh*_ block reverses training-induced SAN bradycardia.

### Training-Induced Bradycardia in Wild-Type Mice Is Due to Remodeling of Intrinsic SAN Automaticity

Consistent with our previous studies in rodent models and in human athletes (D’Souza et al., 2014, 2017), we found that training regimen affects the sinus rate and the atrioventricular conduction time (Figure 1). We cannot completely exclude that, because of the decrease of expression of Ca_v_1.3 in the SAN, trained mice may present with increased susceptibility to inducible atrial arrhythmia. However, we have previously reported that ablation of *I*_*KACh*_ prevents arrhythmias in *Ca_*v*_1.3^–/–^* mice (Mesirca et al., 2016a). Ablation of *I*_*KACh*_ concomitantly rescued SAN automaticity and atrioventricular conduction (Figure 1). We have shown that *Girk4*^–/–^ mice have reduced HF and LF integrals of the HRV spectrum (Wickman et al., 1998; Mesirca et al., 2013). This reduction is arguably due to loss of the fast G protein dependent pathway of HR regulation by the parasympathetic branch of the autonomic nervous system (Wickman et al., 1998). In the present study, despite a reduction in total HRV, we do not find evidence of a differential sympatho-vagal balance in WT and *Girk4*^–/–^ mice. Indeed, the heart rates of WT and *Girk4*^–/–^ mice *in vivo* similarly responded to atropine or propranolol, an observation that indicates that *Girk4*^–/–^ mice do not present with sympathetic or parasympathetic overdrive secondary to global *Girk4* knockout (Mesirca et al., 2013). Consistent with these previous results, the training regimen did not affect the rate independent HRV parameters of HR of WT and *Girk4*^–/–^ mice. Indeed, training did not affect either the SDNN or the LF/HF ratio of HR of WT and *Girk4*^–/–^ mice, suggesting similar degrees of vagal input in the two mouse strains (Figure 2 and Supplementary Figure 5). However, the training regimen augmented the power spectral density in WT but not in *Girk4*^–/–^ mice (Figure 2). Although this could be argued to be evidence of a change in autonomic innervation following training, HRV has been shown to be strongly influenced by HR (Zaza and Lombardi, 2001; Monfredi et al., 2014; Dias da Silva et al., 2015), and the training-induced increase in PSD in WT mice could be the result of the concomitant decrease in HR. Taken together, the effects of the training regimen on HRV, together with our observation that the difference between the HR recorded in sedentary and trained WT mice is maintained after pharmacologic inhibition of autonomic nervous system input, show that intrinsic remodeling of SAN automaticity mediated by regulation of expression of ion channels involved in pacemaking is the predominant mechanism of HR adaptation to training. Consequently, the absence of reduction in *I*_f_, *I_*CaL*_, I*_*CaT*_ with accompanying transcriptional remodeling in *Girk4*^–/–^ mice is unlikely to be due to a differential degree of vagal input in the two mouse strains.

Our previous findings in human athletes (D’Souza et al., 2017) and that of others (Lewis et al., 1980; Katona et al., 1982; Maciel et al., 1985; Dickhuth et al., 1987; Stein et al., 2002) demonstrated intrinsic HR slowing accompanied training-induced bradycardia. These findings are contested by studies in rodents (e.g., Aschar-Sobbi et al., 2015) and dogs (Billman et al., 2015) where training induced intrinsic slowing was not observed and instead a role for high vagal tone was determined. The evidence for intrinsic vs. autonomic mechanisms in underlying training-induced sinus bradycardia has been extensively reviewed by our group (Boyett et al., 2013, 2017; D’Souza et al., 2015, 2019) and we posit that non-uniform methodology (species, drug doses, and training modalities) contribute to the reported discrepancies. Furthermore, based on our present results we advance that both vagally mediated and intrinsic SAN remodeling-based mechanisms of the HR adaptation to training may be reconciled: we speculate that (currently uncharacterized) training-induced alterations in sympathetic and/or parasympathetic input may trigger transcriptional remodeling of SAN ion channels, leading to a decrease in intrinsic automaticity as observed. This hypothesis is consistent with one of the main observations of the present study, that genetic deletion of *I*_*KACh*_, an important downstream effector of the parasympathetic nervous system, prevents training-induced remodeling of SAN automaticity. Our model of training induced secondary SAN bradycardia differs from the one used by Long et al. of a canine model of SAN dysfunction associated with heart failure (Long et al., 2020). The pathological mechanisms underlying secondary SAN dysfunction can differ among forms, so the role of *I*_*KACh*_. Indeed, while it appears that remodeling of GIRK1 and GIRK4 expression is an important pathophysiological mechanism in SAN dysfunction associated with heart failure (Long et al., 2020), we did not find evidence for *I*_*KACh*_ remodeling by the training regimen. However, these studies combined underscores the importance of *I*_*KACh*_ has an important physiopathological mechanism in several forms of SAN dysfunction.

### Genetic Ablation of *I*_*KACh*_ Prevents Training-Induced Remodeling of SAN Ion Channels Involved in Automaticity

The observation that genetic ablation of *I*_*KACh*_ prevented training induced remodeling of *I*_f_, *I*_*CaT*_ and *I*_*CaL*_ is striking. Indeed, it could have been expected that *I*_*KACh*_ abolition would have compensated for decrease in inward ionic currents involved in pacemaking, thereby leading to a lack of net effect on pacemaking by the training regimen. We previously described this mechanism of diastolic inward/outward current balance in mice lacking *I*_f_ conductance or after genetic ablation of Ca_v_1.3 and Ca_v_3.1 channels (Mesirca et al., 2014, 2016a; Bidaud et al., 2020). This compensatory effect of acute *I*_*KACh*_ inhibition was observed also in this study in trained WT mice (Figure 1E), in which three important inward currents contributing to diastolic depolarization are downregulated (Figures 4, 5). The mechanism linking constitutive loss of *I*_*KACh*_ to suppression of protein downregulation or miR-mediated ion channel remodeling (Figures 6, 7) is at present unknown. However, it raises some important issues. Indeed, it indicates that *Girk4* knockout has complex transcriptional consequences for HCN4 and its repressor miRs. Such a role for *Girk4* is surprising and new. Could *I*_*KACh*_ channels be important signaling molecules? As a parasympathetic nervous system effector, it is at the interface of the vagus and the heart and this could be an important position for a signaling molecule. An attractive possibility is that GIRK4 may have nuclear functions as a transcription factor, as has been previously described for cardiac KCHIP2, the accessory subunit defining *I*_*to,f*_ (Nassal et al., 2017). Alternatively, GIRK4 could be a key signaling hub wherein genetic ablation of *I*_*KACh*_ *per se*, or deletion of GIRK4 from the plasma membrane, blocks a signaling pathway that triggers miR-mediated remodeling of the expression of a particular set of genes including *Hcn4*. Another intriguing development from this work is the differential regulation of HCN4 vs. the L- and T-type calcium channels in response to training. Whereas *Hcn4* downregulation explains *I*_f_ reduction in wild-type animals, reduced *I*_*CaT*_ and *I*_*CaL*_ could not be attributed to reduced expression of mRNAs coding for pore forming Ca^2+^ channel α1 subunits (Figure 7) or accessory subunits of voltage-gated Ca^2+^ channels (Supplementary Figure 9). Whether the observed reduction in Ca_v_1.3 and its restoration on *Girk4* knockout is due to (albeit less well established) miR-mediated translational inhibition without mRNA degradation (Eulalio et al., 2007) or other ion channel modulators such as protein kinases merits further study.

### Training-Induced Slowing of SAN Cells Pacemaker Activity Is Due to Downregulation of *I*_f_, *I*_*CaT*_ and *I*_*CaL*_

In the present study, we report that *I*_f_, *I*_*CaL*_ and *I*_*CaT*_ amplitudes are diminished by the training regimen (Figures 4, 5). In addition, training positively shifted *I*_*CaL*_ half-activation (Supplementary Figure 6). While this study is consistent with previous work showing that training induces *I*_f_ down-regulation (D’Souza et al., 2014), the role of *I*_*CaT*_ and *I*_*CaL*_ in mediating training induced bradycardia is an emerging one. We showed previously that Ca_*v*_3.1-mediated *I*_*CaT*_ contributes to pacemaker activity of adult mouse SAN cells. Genetic ablation of Ca_*v*_3.1-mediated *I*_*CaT*_ reduced basal automaticity of SAN cells by 16% in comparison to wild-type counterparts (Baudot et al., 2020), a value that is comparable to the predicted 13% slowing of basal automaticity, calculated by reducing *I*_*CaT*_ maximal conductance of 30%, as observed experimentally (Figure 5 and Supplementary Figure 11). SAN cells express distinct L-type Ca^2+^ channel isoforms, Ca_*v*_1.3 and Ca_*v*_1.2 (Mangoni et al., 2003; Marionneau et al., 2005). One of the key differences between Ca_*v*_1.3- and Ca_*v*_1.2-mediated *I*_*CaL*_ is their voltage-dependence for activation (Mangoni et al., 2006a). Ca_*v*_1.3-mediated *I*_*CaL*_ activates at negative voltages and contributes to the generation of diastolic depolarization by supplying inward current (Mangoni et al., 2003; Toyoda et al., 2017) and controlling diastolic RyR-dependent Ca^2+^ release during pacemaking (Torrente et al., 2016). Ca_*v*_1.2-mediated *I*_*CaL*_ activates at more positive voltages than Ca_*v*_1.3, contributes to the action potential upstroke phase and to regulation of SR Ca^2+^ load (Torrente et al., 2016). Because of the importance of Ca_*v*_1.3-mediated *I*_*CaL*_ in the generation of diastolic depolarization, we may expect that the decrease in the amplitude of *I*_*CaL*_ induced by the training regimen would lead to slowing of SAN spontaneous activity. Our numerical simulations predict that a decrease of 48% of *I*_*CaL*_ peak density and a 4 mV positive shift in activation does not prolong the calculated cycle length (Supplementary Figure 11). This inconsistency could be explained, in part by predicted shortening of action potential duration following decreased *I*_*CaL*_ magnitude (Figure 8). A similar prediction was reported by Zhang et al. in modeling the negative chronotropic effect of ACh in the central part of the rabbit SAN (Zhang et al., 2002). However, our model does predict prolongation of the diastolic interval by concomitant *I*_*CaL*_ down-regulation and positive shift of *I*_*CaL*_ activation (Supplementary Figure 11), in line with the importance of Ca_v_1.3-mediated *I*_*CaL*_ in the generation pacemaker activity. In particular, our model predicts slowing of diastolic interval by positive shifting the voltage for half activation of Ca_*v*_1.3-mediated *I*_*CaL*_ (Supplementary Figure 13). Furthermore, our recent work showed that Ca_*v*_1.3 is an essential molecular determinant of the sustained inward current *I*_*st*_ (Toyoda et al., 2017). In addition, the density of *I*_*st*_ positively correlates with that of *I*_*CaL*_ and mRNA coding for Ca_*v*_1.3 in guinea-pig SAN cells (Toyoda et al., 2018). It is thus possible that since the training regimen down-regulated Ca_*v*_1.3, this could also have affected also *I*_*st*_ density, further contributing to slowing of automaticity. Even if our model predicts slowing of diastolic depolarization when *I*_*st*_ is reduced (data not shown), we did not include this in our calculations, as we did not directly measure the effects of training regimen on *I*_*st*_ expression in this study.

When training-induced changes in *I*_f_, *I*_*CaT*_ and *I*_*CaL*_ are combined, the predicted magnitude of slowing is higher than the sum of the predicted individual contributions of *I*_f_, *I*_*CaT*_ and *I*_*CaL*_ (Figure 8 and Supplementary Figure 11), which suggests a non-linear quantitative impact of predicted loss of the cell depolarization reserve. This is consistent with our recent observation that the relative HR slowing observed after administration of the *I*_f_ inhibitor ivabradine is higher in mice lacking both Ca_v_1.3 and Ca_v_3.1 (*Ca_*v*_1.3^–/–^/Ca_*v*_3.1^–/–^*) channels, than in wild-type counterparts (Baudot et al., 2020). Previous work showed that administration of the *I*_f_ inhibitor ivabradine negated the difference in HR between sedentary and trained mice (D’Souza et al., 2014). However, the present study does not contradict these data. Indeed, we cannot exclude that, while *I*_f_, *I*_*CaT*_ and *I*_*CaL*_ jointly contribute to slowed cellular automaticity *ex vivo* conditions (Figure 3), the permanent action of the autonomic nervous system and mechanical hemodynamic forces regulating SAN activity and HR *in vivo*, modulates the relative contribution of HCN4, Ca_v_3.1 and Ca_v_1.3 to pacemaking in trained animals. Moreover, previous work in rabbit SAN cells has showed that *I*_f_ block by ivabradine reduces diastolic RyR-dependent Ca^2+^ release (Yaniv et al., 2013). It has thus been proposed that *I*_f_ inhibition slows pacemaker activity not only via a reduction in inward current via funny channels, but also via reduction in the speed of the Ca^2+^ clock/NCX1 pacemaker mechanism (Yaniv et al., 2013). This proposal would be consistent also with the hypothesis that slowing of diastolic depolarization by ivabradine alters the kinetics of recruitment of *I*_*CaT*_ and Ca_v_1.3-mediated *I*_*CaL*_ in the pacemaker potential range, thereby indirectly contribute to the effect of ivabradine on pacemaker activity *in vitro* and on HR *in vivo*. In conclusion, our study indicates that the training regimen slows the HR via regulation of three ionic currents important for pacemaker activity: *I*_f_, *I*_*CaT*_ and *I*_*CaL*_. Future studies will be required to understand how these currents interact to generate pacemaking, mechanisms that current models of automaticity cannot reproduce fully.

## Conclusion

Athletes are considered to be part of the healthiest fraction of the population. However, in the long term there is emerging evidence for SAN dysfunction leading to an increased incidence of electronic pacemaker implantation in this population (Baldesberger et al., 2008). For the first time, our studies show that genetic targeting of *I*_*KACh*_ is an effective strategy to control remodeling of ionic currents and sinus bradycardia in a murine model of training-induced SAN dysfunction. Gene therapy or pharmacological targeting of *I*_*KACh*_ may therefore represent a viable alternative to pacemaker implantation for the management of pathological bradyarrhythmias seen in some veteran athletes.

## Data Availability Statement

The raw data supporting the conclusions of this manuscript will be made available by the authors, without undue reservation, to any qualified researcher.

## Ethics Statement

The animal study was reviewed and approved by Ethical committee of the University of Montpellier and the French Ministry of Agriculture (protocol no: 2017010310594939).

## Author Contributions

PM, IB, AD’S, KW, MB, and MM designed the research and wrote the manuscript. PM, IB, AD’S, GF, ET, DG, ST, CA, AC, AT, JR, and MM performed the experiments. PM, IB, AD’S, ET, MB, and MM analyzed the results. All authors contributed to the article and approved the submitted version.

## Conflict of Interest

The authors declare that the research was conducted in the absence of any commercial or financial relationships that could be construed as a potential conflict of interest.

## Footnotes

1 http://nsr.bioeng.washington.edu/jsim/
