# Supplementary material for: Genetic Ablation of G Protein-Gated Inwardly Rectifying K+ Channels Prevents Training-Induced Sinus Bradycardia
Source: Front Physiol. 2021 Jan 20;11:519382. doi: 10.3389/fphys.2020.519382 (PMC7857143; doi:10.3389/fphys.2020.519382)
Supplement: Supplementary file 1 [file Data_Sheet_1.PDF]

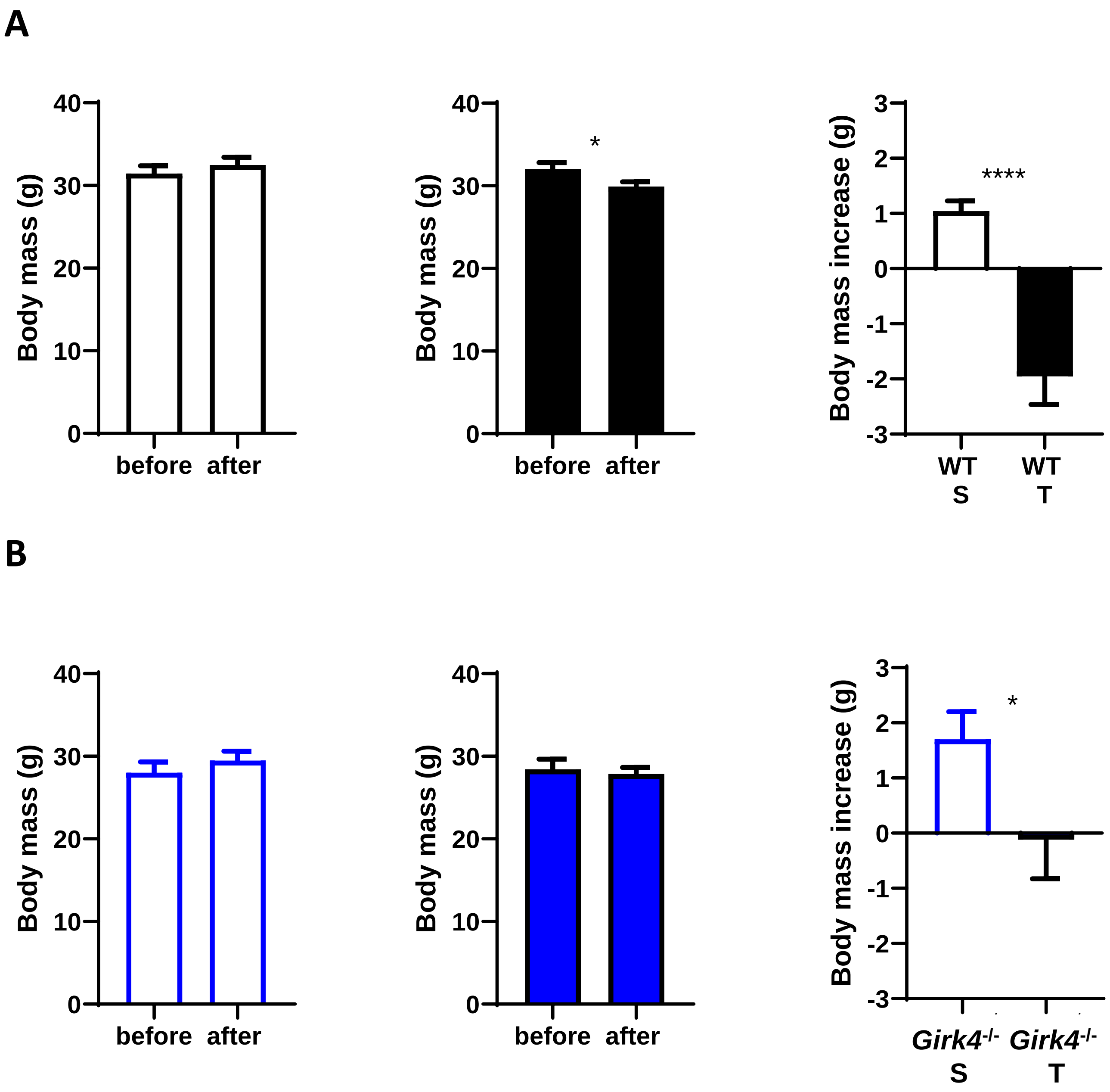

Supplementary Figure 1. (A) Body weight measured in WT sedentary (empty bars) and WT trained (filled bars) group before and after the period of exercise (training), or sham-exercise (sedentary). (A, right panel) Difference between body mass measured before training and at the end of the training regimen in WT sedentary (WT S) and WT trained (WT T) animals. (B). Same as in (A) but in *Girk4*$^{-/-}$ mice. Statistics: unpaired Student's t-test. Error bars indicate s.e.m. *$p<0.05$, ****$p<0.0001$.

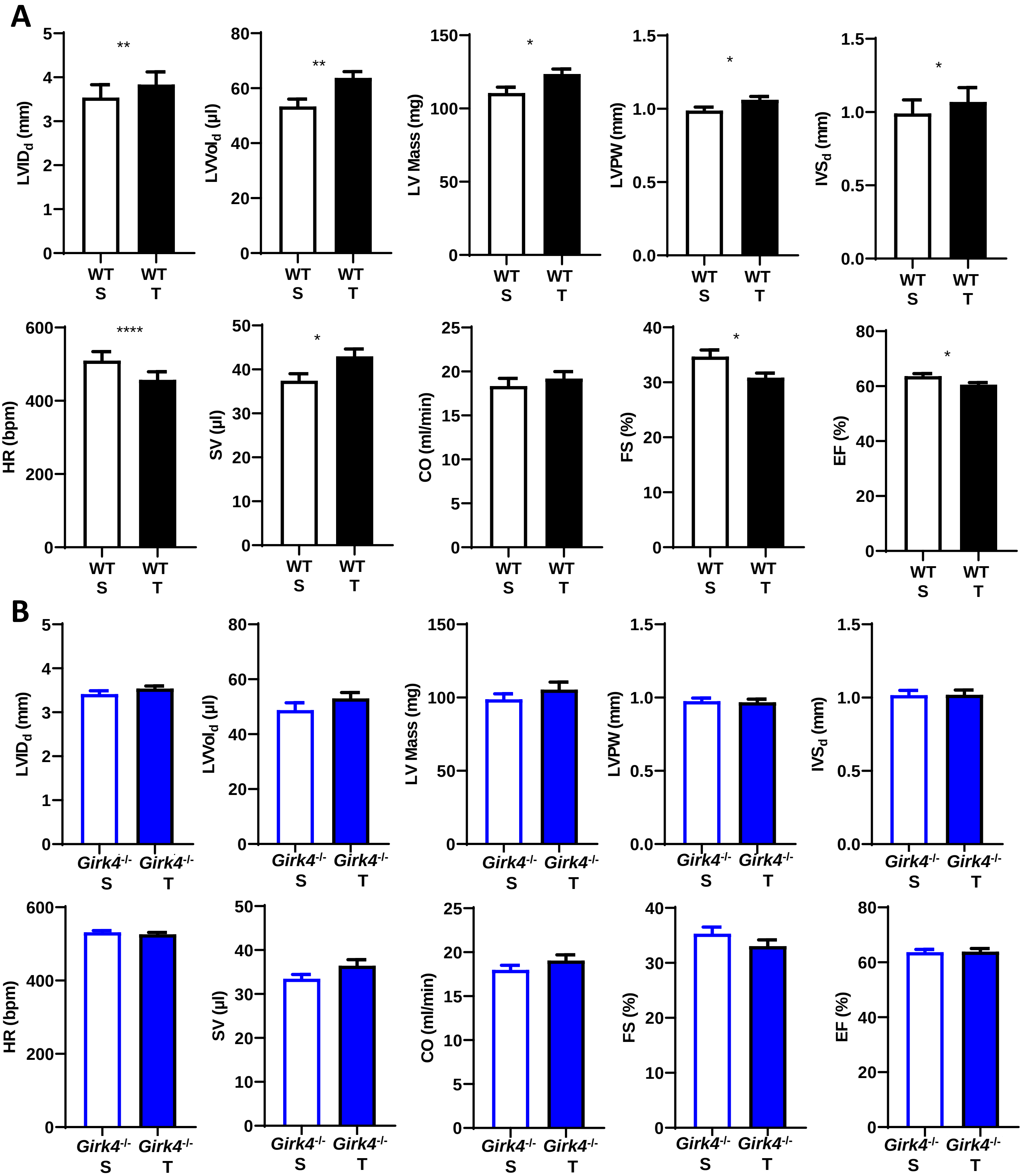

Supplementary Figure 2. Echocardiographic analysis of left ventricular size and function in sedentary (empty bars) and trained (filled bars) WT (A) and *Girk4*$^{-/-}$ (B) mice. Statistics: unpaired Student's t-test. Error bars indicate s.e.m. *$p<0.05$, **$p<0.01$, ****$p<0.0001$. LVIDd: Left Ventricular Internal Diameter (diastole); LVVold: Left Ventricular Volume (diastole); LV Mass: Left Ventricular Mass; LVPW: Left Ventricular Posterior Wall (diastole); IVSd: Inter-ventricular septum (diastole); SV: Stroke Volume; CO: Cardiac Output; FS: Fractional Shortening; EF: Ejection Fraction.

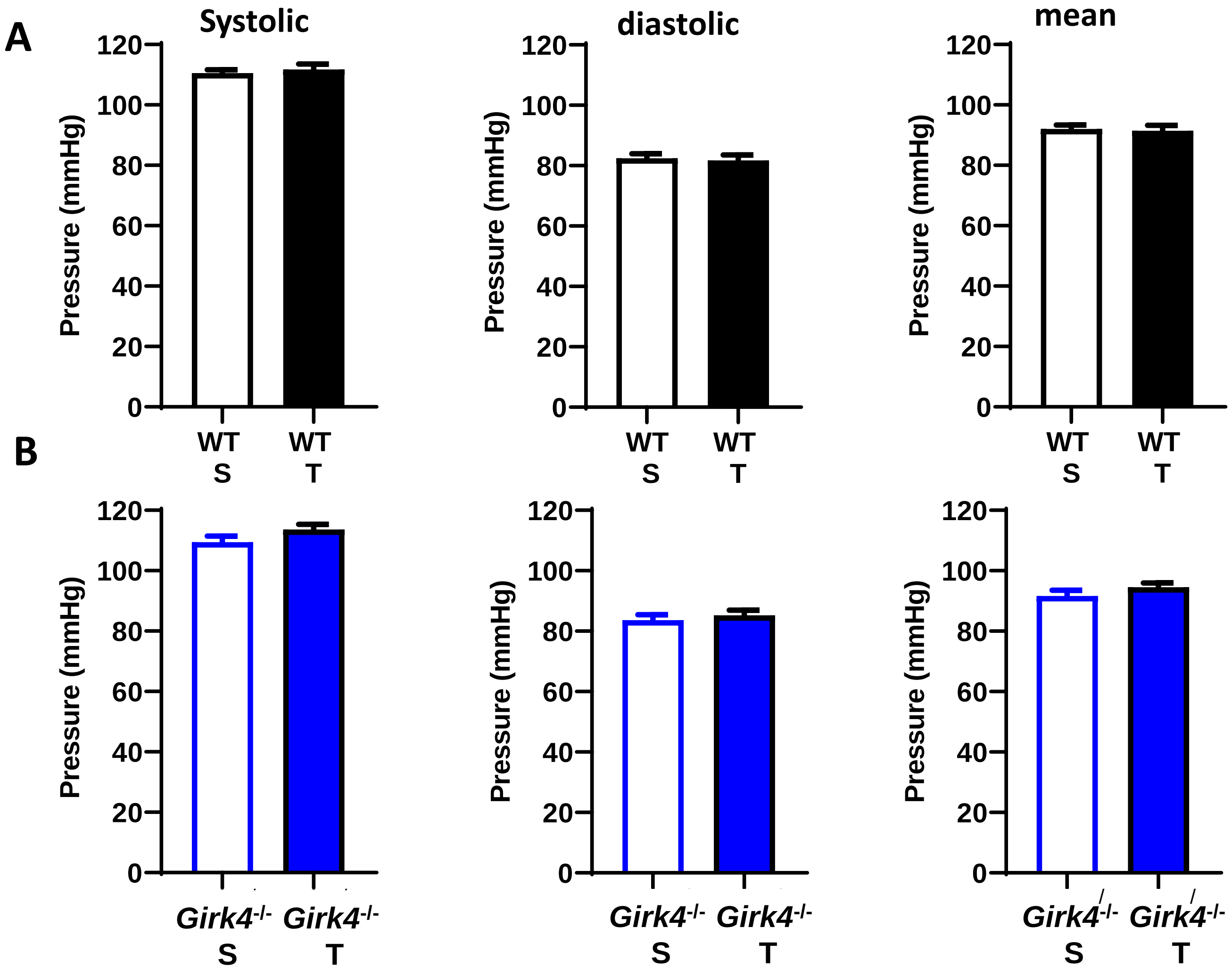

Supplementary Figure 3. Systolic (left), diastolic (center), and mean blood pressure (right) in sedentary (empty bars) and trained (filled bars) WT (A) and *Girk4*$^{-/-}$ (B) mice. Statistics: unpaired Student's t-test. Error bars indicate s.e.m..

Suppelmentary Dataset 1: Telemetric measurements of heart rate (HR), PR intervals and PSD in sedentary wild-type and *Girk4*$^{-/-}$ mice compared by gender. Statistics: unpaired t-test.

HR WT : 546±6 vs 548±8 for females and males respectively, p=0.84.
HR *Girk4*$^{-/-}$: 591±10 vs 574±5 for females and males respectively, p=0.19.
PR WT : 34±1 vs 34±1 for females and males respectively, p=0.96.
PR *Girk4*$^{-/-}$: 33±1 vs 33±2 for females and males respectively, p=0.92.
PSD WT : 4.2±0.1 vs 4.0±0.1 for females and males respectively, p=0.31.
PSD *Girk4*$^{-/-}$: 3.1±0.2 vs 3.4±0.2 for females and males respectively, p=0.50.

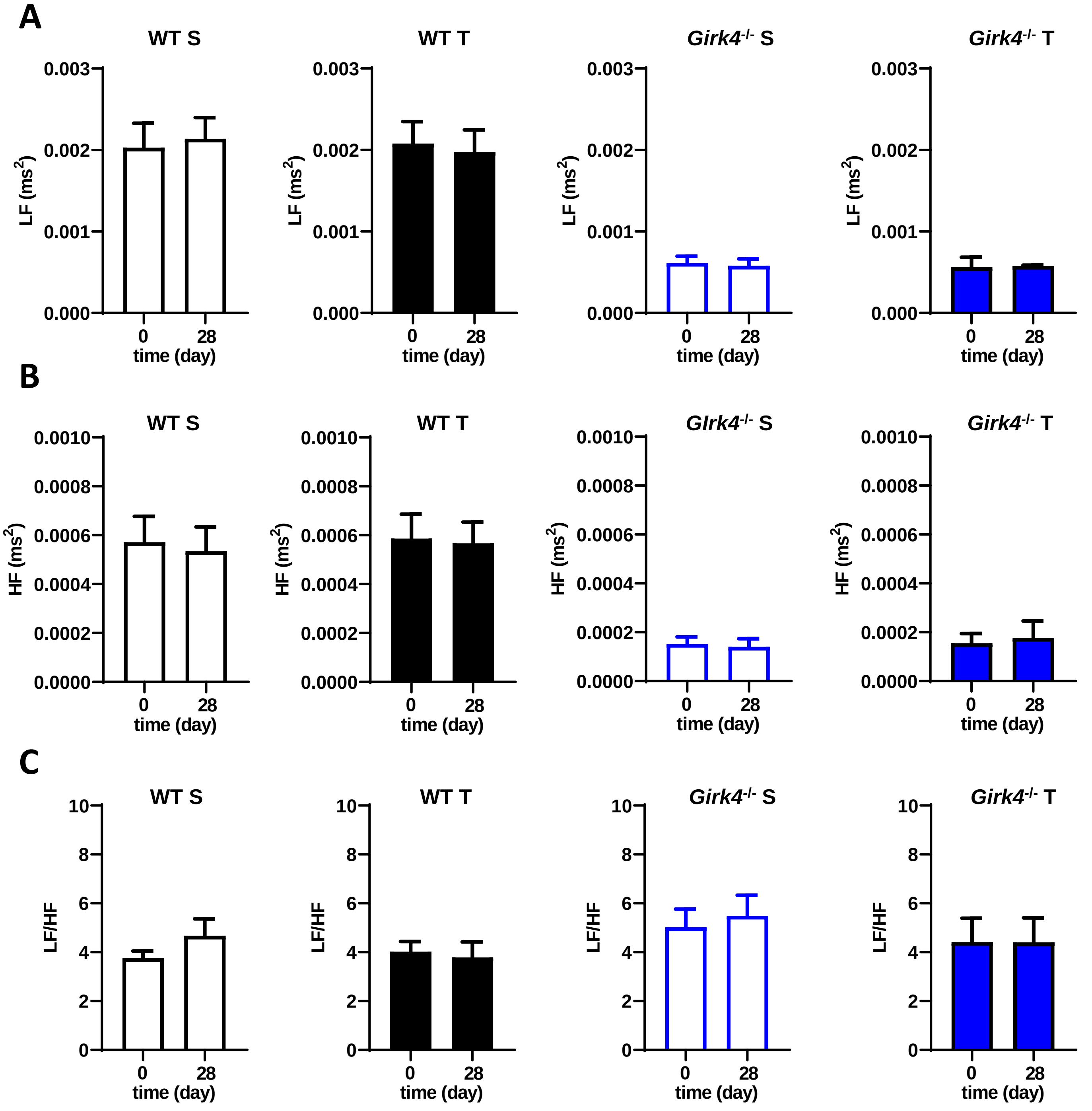

Supplementary Figure 4. Heart rate variability (HRV) frequency-domain analysis measured over 5-min stable ECG recording in sedentary (open bars, S) and trained (filled bars, T) wild-type (black, WT) and *Girk4*$^{-/-}$ (blue) mice at day 0 and at day 28. (A) Low frequency (LF) spectra (0.15-1.5 Hz). (B) high-frequency (HF) spectra (1.5-5 Hz) and (C) ratio between LF and HF values. Statistics: unpaired Student's t-test. Error bars indicate s.e.m..

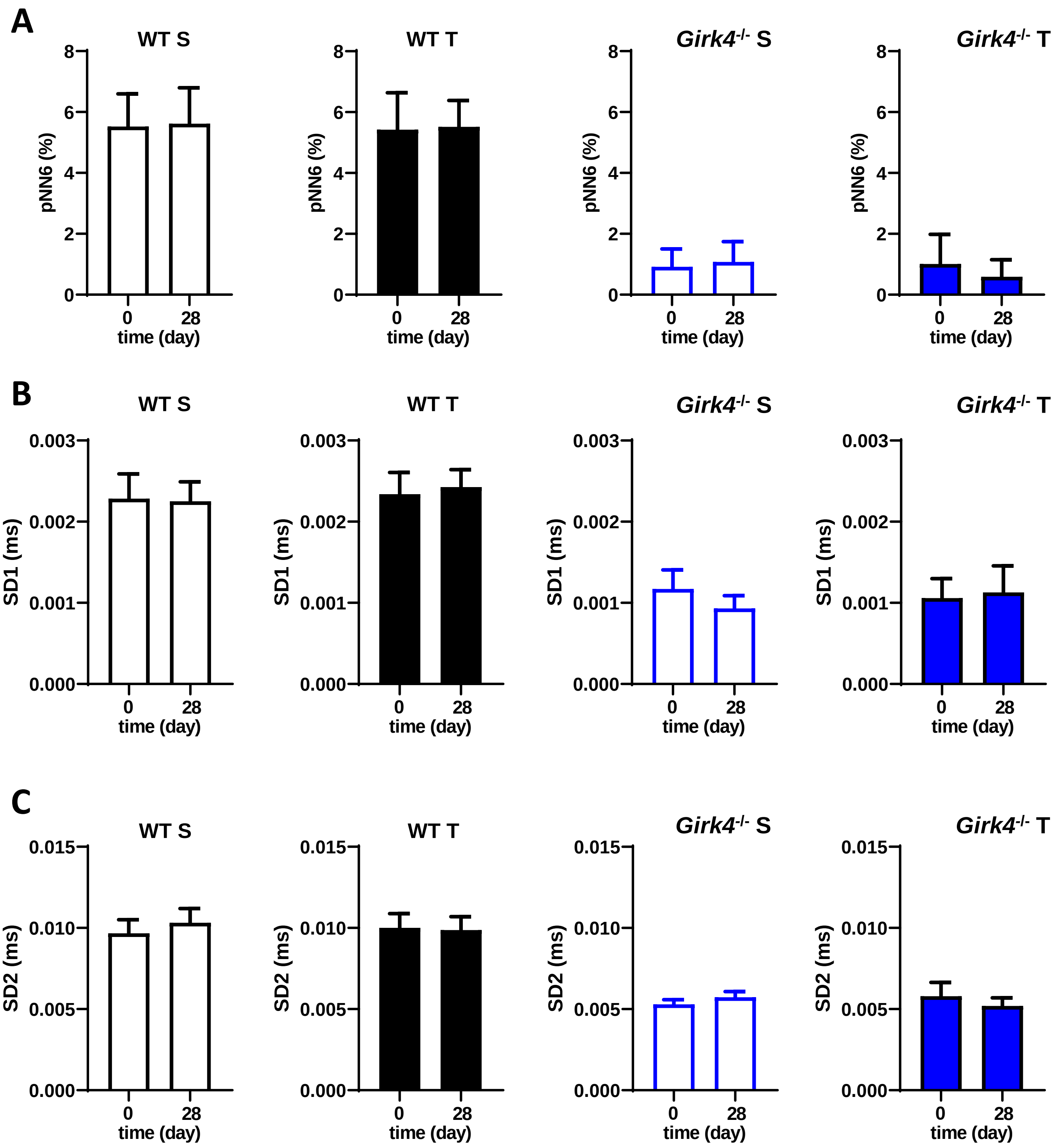

Supplementary Figure 5. Heart rate variability (HRV) analysis measured over 5-min stable ECG recordings in sedentary (open bars, S) and trained (filled bars, T) wild-type (black, WT) and *Girk4*$^{-/-}$ (blue) mice at day 0 and at day 28. (A) percentage of consecutive R-R intervals differing by >6 ms (pNN6). Standard deviation of instantaneous beat-to-beat interval variability (SD1, B) and continuous long-term R-R interval variability (SD2, C) provided by ellipse-fitting technique of the Poincaré scatter-gram. Statistics: unpaired Student's t-test. Error bars indicate s.e.m..

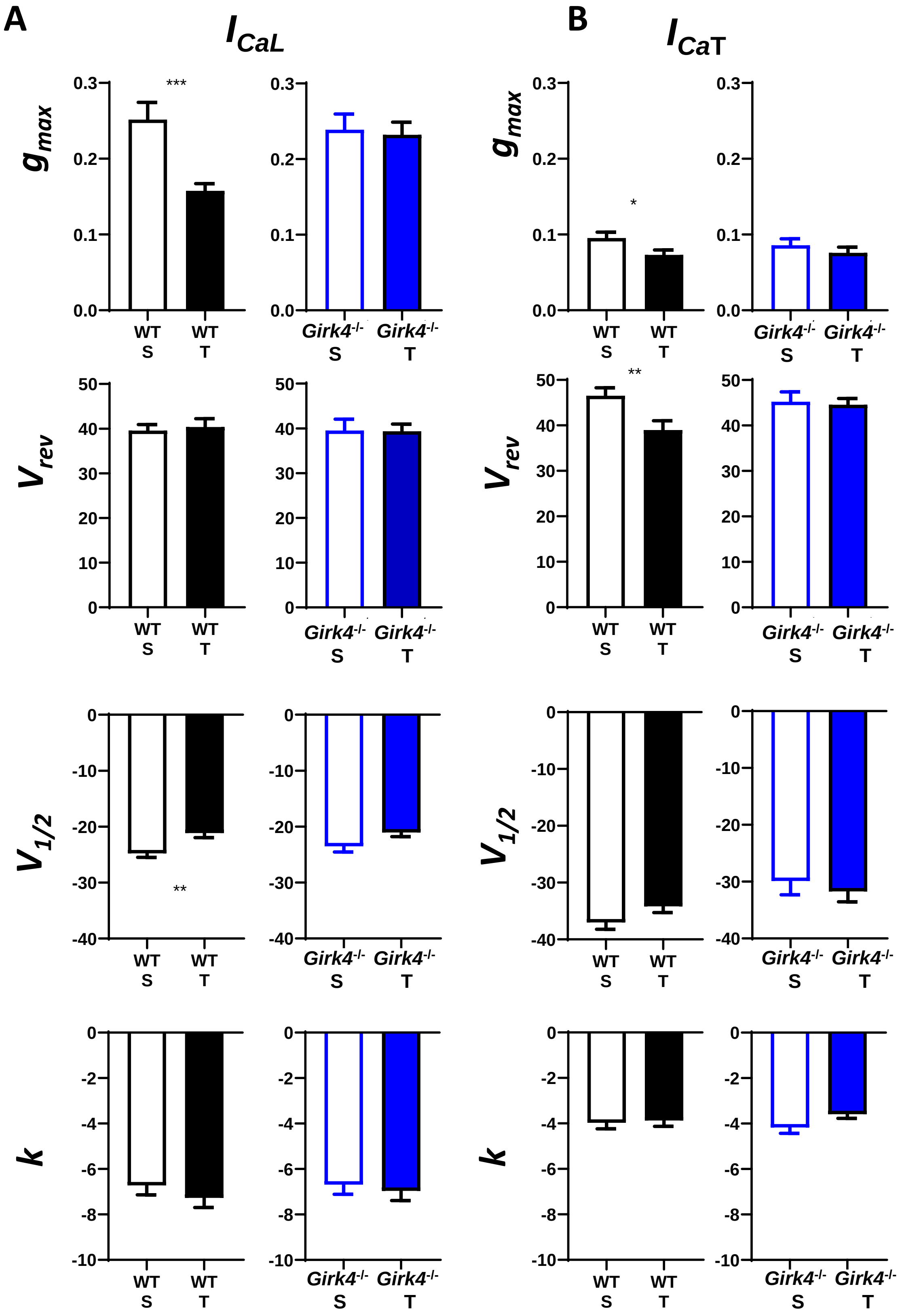

Supplementary Figure 6. L-type (I_{CaL}, A) and T-type (I_{CaT}, B) Ca^{2+} maximal conductance (g_{max}), apparent reversal potential (*Vrev*), half-activation voltages ($V_{1/2}$), and activation slope factor (*k*), calculated from I-V curves fitted with a modified Boltzmann equation (see Methods). Statistics: one-way analysis of variance. Error bars indicate s.e.m.. WTS: WT sedentary; WTT: WT trained; *Girk4*$^{-/-}$ S: *Girk4*$^{-/-}$ sedentary and *Girk4*$^{-/-}$ T: *Girk4*$^{-/-}$ trained.

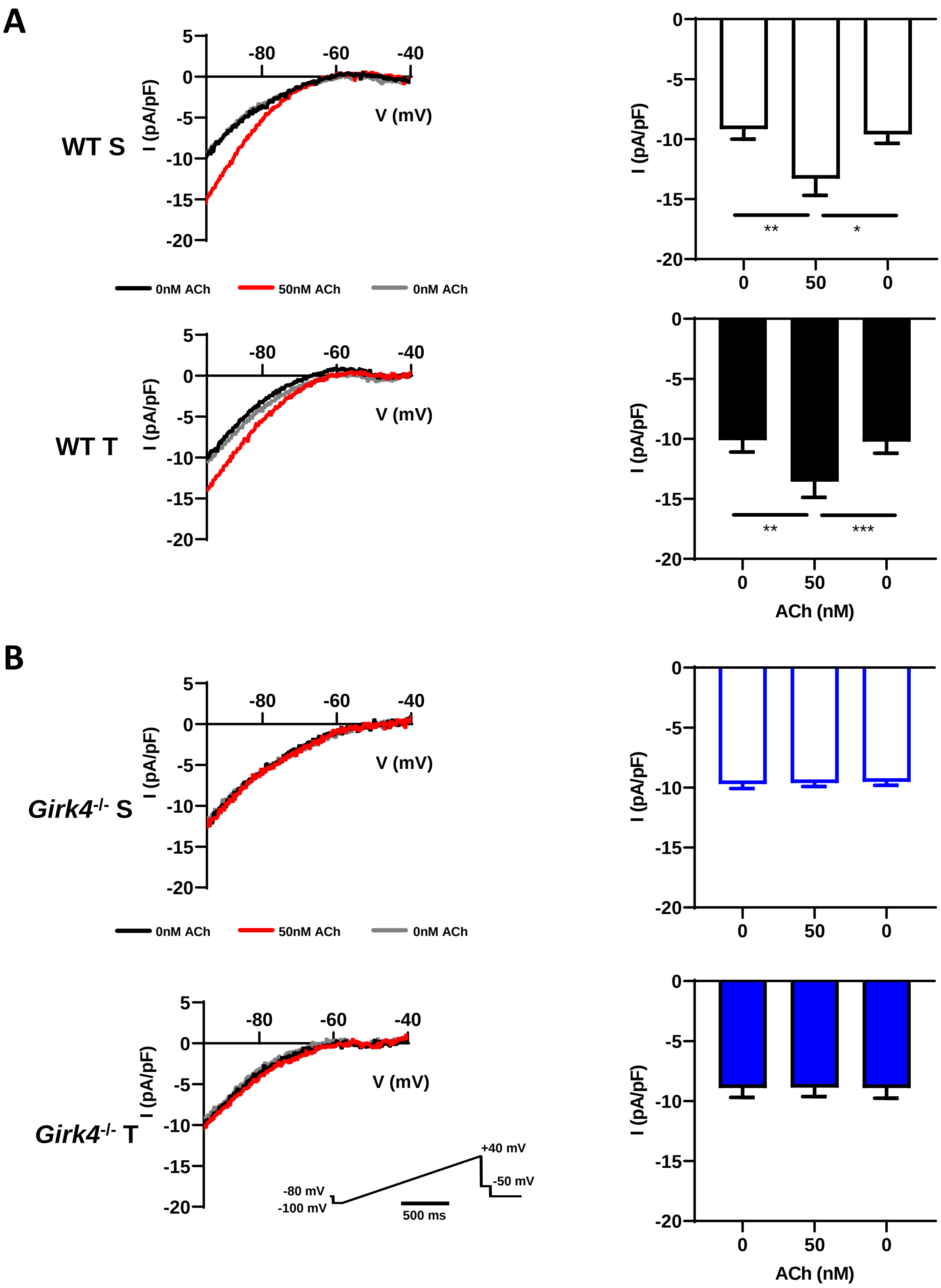

Supplementary Figure 7. Sample traces of I_{KACh} before (black line), during (red line) and after (gray line) 50 nM ACh perfusion in SAN myocytes of sedentary (top left) and trained (bottom left) WT mice (A) and *Girk4*$^{-/-}$ (B) mice. Averaged I_{KACh} density recorded in SAN cells from sedentary (top right, empty bars) and trained (bottom right, filled bars) WT (A) and *Girk4*$^{-/-}$ (B) mice recorded before, during and after ACh 50nM perfusion. (B, inset). Voltage clamp protocol. Statistics: one-way analysis of variance followed by Tukey multiple comparisons test. Error bars indicate s.e.m. *p<0.05, **p<0.01, ***p<0.001.

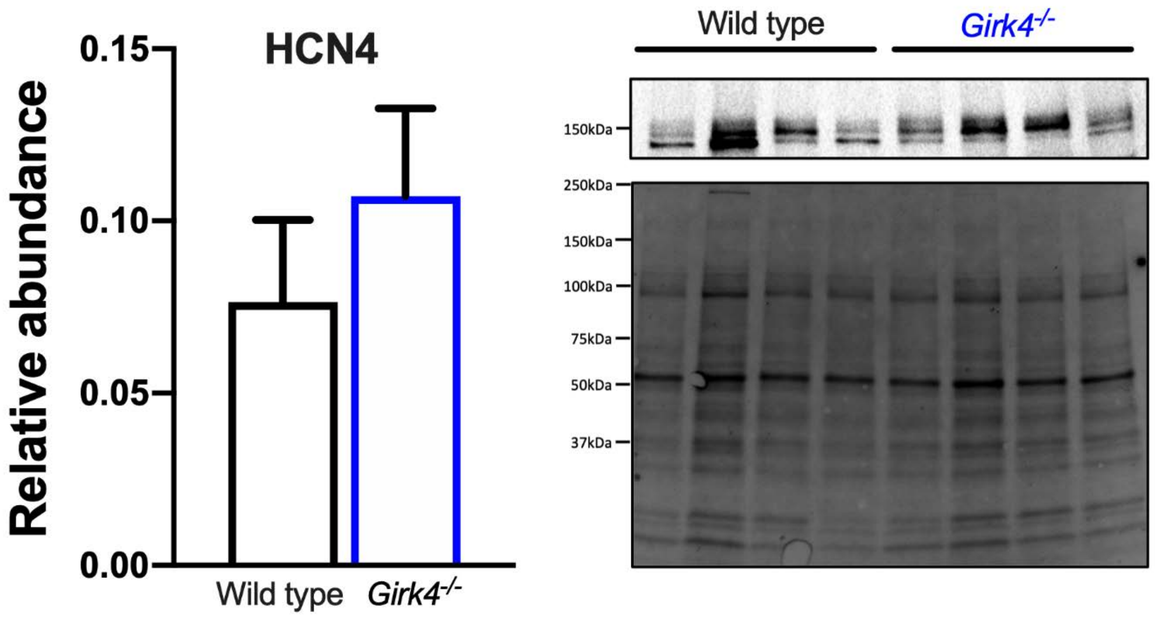

Supplementary Figure 8. Protein expression determined by western blot in individual SAN biopsies isolated from WT sedentary (n=4) and *Girk4*$^{-/-}$ mice sedentary (n=5). Representative western blot with corresponding stain-free total protein gel used for quantification shown in left panel..

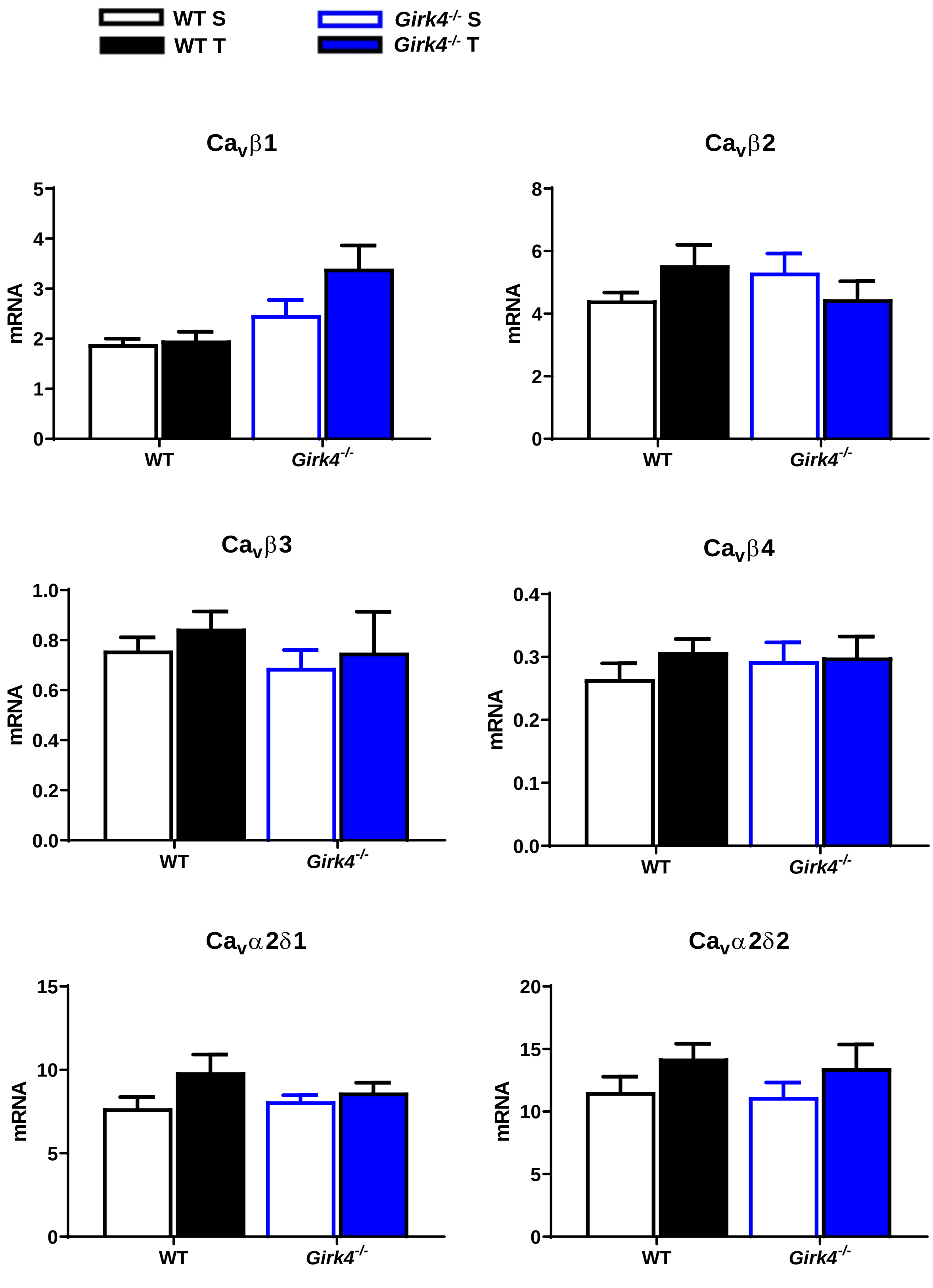

Supplementary Figure 9. mRNA expression of L-type Ca^{2+} channels β and α2–δ subunits in wild-type (WT) and *Girk4*$^{-/-}$ tissues at the end of training (T), or sham-training (S), protocol. Statistics: two-way analysis of variance. Error bars indicate s.e.m..

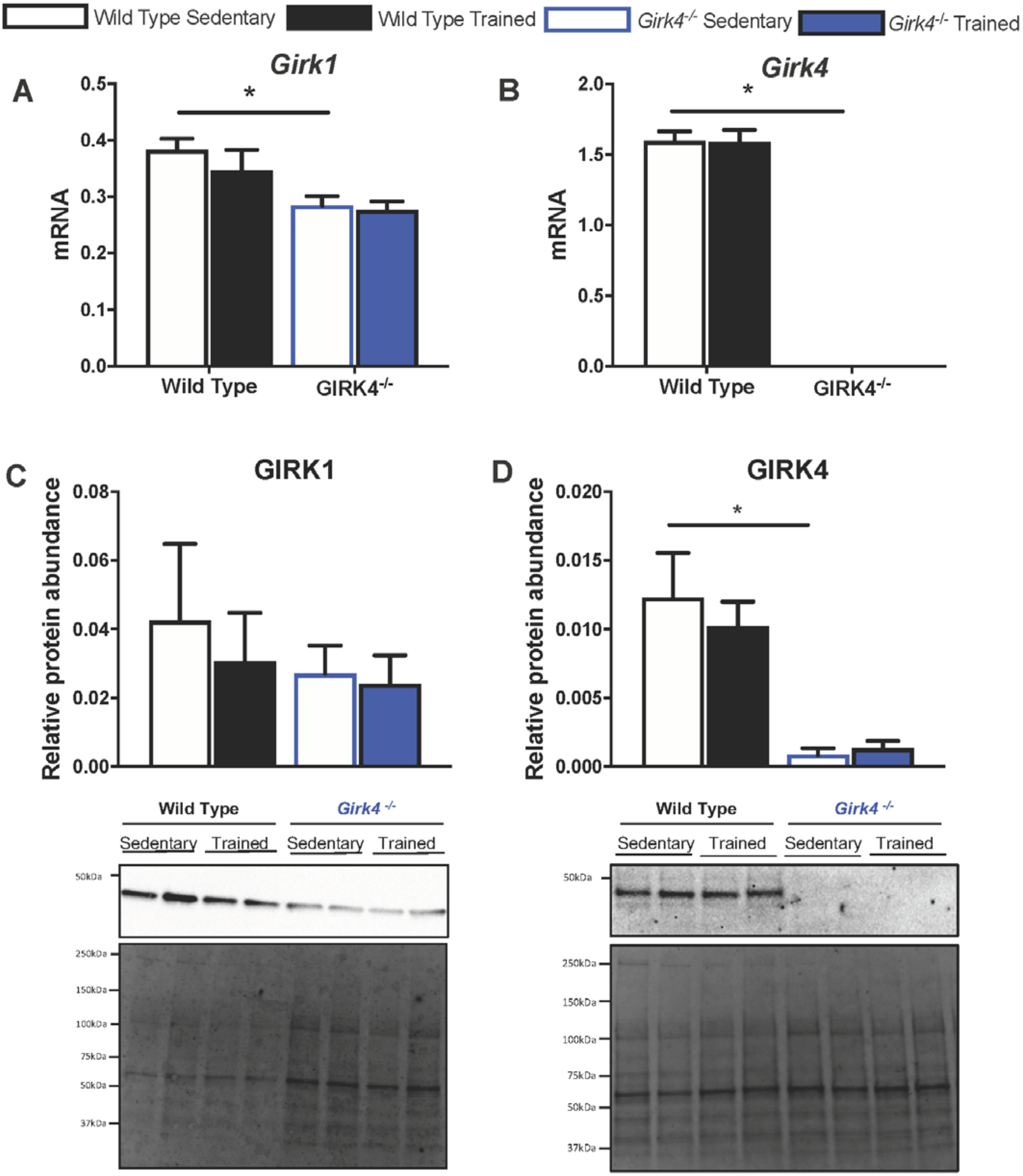

Supplementary Figure 10. mRNA expression of Girk1 (A) and Girk4 (B) in SAN biopsies from WT sedentary (n=10), WT trained (n=10), *Girk4*$^{-/-}$ sedentary (n=9) and *Girk4*$^{-/-}$ trained (n=10) mice. Protein expression determined by western blot using antibodies directed against GIRK1 (C) and GIRK4 (D) in individual sinus node biopsies isolated from sedentary WT (n=4), trained WT (n=4), sedentary *Girk4*$^{-/-}$ mice (n=4) and trained *Girk4*$^{-/-}$ mice (n=4). Representative western blots with corresponding stain-free total protein gel used for quantification shown in lower panel. *$p<0.05$, 2-way ANOVA with Sidak's multiple comparisons test.

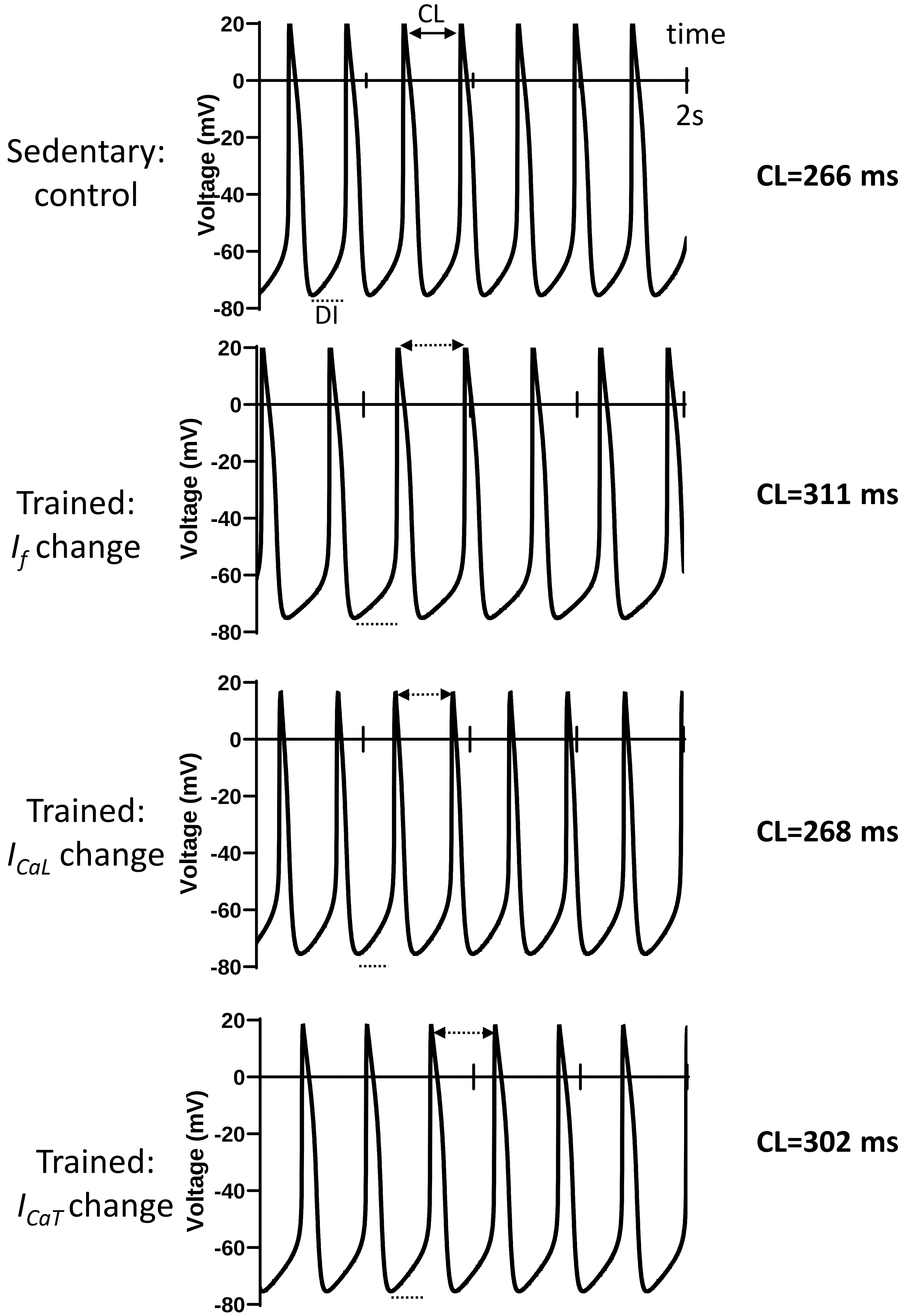

Supplementary Figure 11. Numerical simulations of the predicted impact on SAN cells firing in trained wild-type SAN cells of changes in individual currents. CL, cycle length; DI, diastolic interval.

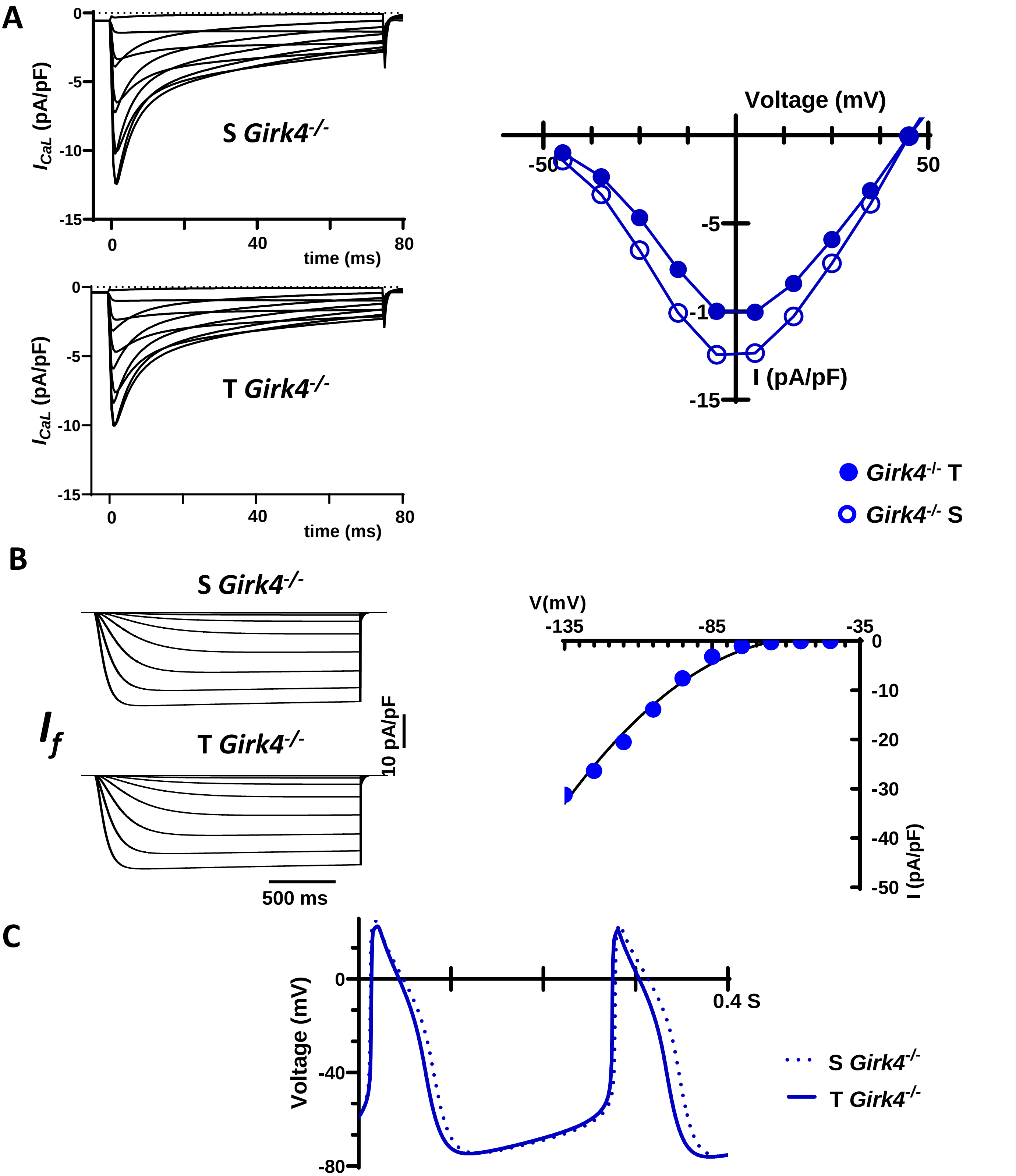

Supplementary Figure 12. Numerical simulation of I_{CaL} (A) and I_f (B) and corresponding predicted I-V curves calculated using values of conductance measured in isolated SAN myocytes from sedentary (S) and trained (T) *Girk4*$^{-/-}$ mice at day 28. In I-V curves, open circles represent current density recorded from *Girk4*$^{-/-}$ sedentary SAN cells, and filled circles parameters from trained *Girk4*$^{-/-}$ SAN cells. (C). Comparison between pacemaker activity computed in control sedentary (S) condition and simulated training-dependent (T) changes in I_{CaL}, I_{CaT} and I_f densities in *Girk4*$^{-/-}$ SAN cells.

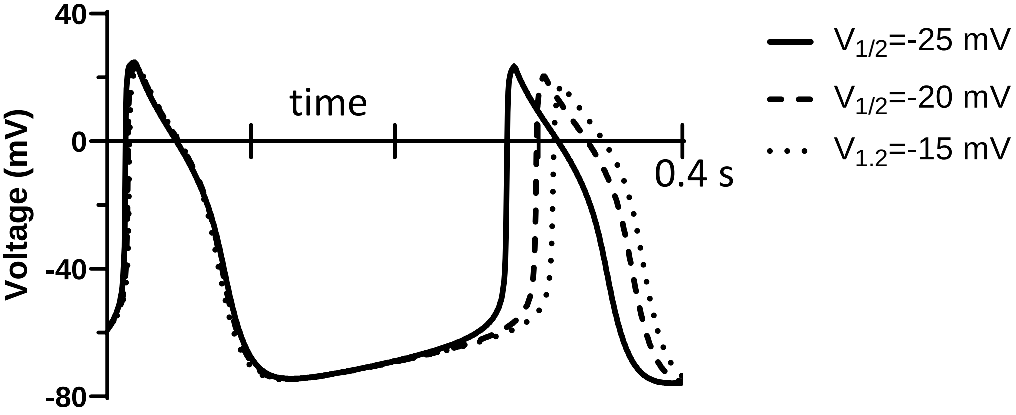

Supplementary Figure 13. Predicted slowing of pacemaker activities calculated using different $Ca_v1.3$-mediated I_{CaL} voltages for half activation ($V_{1/2}$): black line $V_{1/2}$=-25 mV (sedentary condition); dashed line $V_{1/2}$ = -20 mV and dotted line $V_{1/2}$ = -15 mV.
