# Supplementary material for: Genetic Ablation of G Protein-Gated Inwardly Rectifying K+ Channels Prevents Training-Induced Sinus Bradycardia
Source: Front Physiol. 2021 Jan 20;11:519382. doi: 10.3389/fphys.2020.519382 (PMC7857143; doi:10.3389/fphys.2020.519382)
Supplement: Supplementary file 2 [file Table_1.docx]

**Supplementary table 1.** ECG intervals values.

| day 0 | **QRS (ms)** | **QT (ms)** | **QTc (ms)** |  | day 28 | **QRS (ms)** | **QT (ms)** | **QTc (ms)** |
| --- | --- | --- | --- | --- | --- | --- | --- | --- |
| **WT S** | 9.1±0.2 | 40±1 | 39±1 |  | **WT S** | 9.1±0.3 | 40±1 | 37±1 |
| **WT T** | 9.1±0.1 | 41±1 | 39±1 |  | **WT T** | 9.3±0.2 | 43±1 | 39±1 |
| ***Girk4^-/-^* S** | 9.1±0.1 | 42±2 | 41±2 |  | ***Girk4^-/-^* S** | 9.1±0.2 | 42±1 | 40±2 |
| ***Girk4^-/-^* T** | 9.4±0.3 | 44±1 | 43±1 |  | ***Girk4^-/-^* T** | 9.5±0.1 | 40±2 | 38±2 |

Summary table of the ECG interval values recorded in different groups at day 0 and at day 28 (end of protocol). Statistics: two-way analysis of variance. Data are presented as mean ± s.e.m. WT S: WT sedentary; WT T: WT trained; *Girk4^-/-^* S: *Girk4^-/-^* sedentary; *Girk4^-/-^* T: *Girk4^-/-^* trained.
