# Supplementary material for: Genetic Ablation of G Protein-Gated Inwardly Rectifying K+ Channels Prevents Training-Induced Sinus Bradycardia
Source: Front Physiol. 2021 Jan 20;11:519382. doi: 10.3389/fphys.2020.519382 (PMC7857143; doi:10.3389/fphys.2020.519382)
Supplement: Supplementary file 3 [file Table_2.docx]

**Supplementary table 2.** Action potential parameters recorded in isolated SAN cells.

| day 28 | **MDP**  **(mV)** | **Eth (mV)** | **SEDD (mV/ms)** | **dV/dt (mV/ms)** | **APA (mV)** |
| --- | --- | --- | --- | --- | --- |
| **WT S** | -62±1 | -39±1 | 0.44±0.02 | 35±12 | 83±5 |
| **WT T** | -61±1 | -38±1 | 0.35±0.03 | 38±7 | 91±4 |
| ***Girk4^-/-^* S** | -59±1 | -39±1 | 0.42±0.06 | 37±3 | 87±2 |
| ***Girk4^-/-^* T** | -58±1 | -40±1 | 0.43±0.04 | 36±5 | 83±3 |
|  |  |  |  |  |  |
| day 28 | **APD30**  **(ms)** | **APD50 (ms)** | **APD70 (ms)** | **APD90 (ms)** |  |
| **WT S** | 19±2^#^ | 30±3^#^ | 47±3^##^ | 80±5^###^ |  |
| **WT T** | 26±3 | 45±4 | 77±7 | 137±10 |  |
| ***Girk4^-/-^* S** | 16±2^##^ | 25±3^##^ | 42±5^###^ | 80±11^###^ |  |
| ***Girk4^-/-^* T** | 16±1^##^ | 25±1^###^ | 39±2^####^ | 70±5^####^ |  |

# statistical significance versus WT T

Action potential parameters in WT sedentary (WT S), WT trained (WT T), *Girk4^-/-^* sedentary (*Girk4^-/-^* S) and *Girk4^-/-^* trained (*Girk4^-/-^* T) isolated SAN cells at day 28 of protocol. Statistics: one-way analysis of variance followed by Tukey multiple comparisons test. ^#^p<0.05, ^##^p<0.01, ^###^p<0.001, ^####^p<0.0001. Data are presented as mean ± s.e.m..

MDP = maximum diastolic potential; Eth = action potential threshold; SEDD = exponential diastolic depolarization slope; dV/dt = action potential upstroke velocity; APA = action potential amplitude; APD = action potential duration at 30%, 50%, 70% or 90% of repolarization.
